# DNA-Methylation and Body Composition in Preschool Children: Epigenome-Wide-Analysis in the European Childhood Obesity Project (CHOP)-Study

**DOI:** 10.1038/s41598-017-13099-4

**Published:** 2017-10-30

**Authors:** Peter Rzehak, Marcela Covic, Richard Saffery, Eva Reischl, Simone Wahl, Veit Grote, Martina Weber, Annick Xhonneux, Jean-Paul Langhendries, Natalia Ferre, Ricardo Closa-Monasterolo, Joaquin Escribano, Elvira Verduci, Enrica Riva, Piotr Socha, Dariusz Gruszfeld, Berthold Koletzko

**Affiliations:** 1 Division of Metabolic and Nutritional Medicine, Dr. von Hauner Children’s Hospital, Ludwig-Maximilians Universität München (LMU), Munich, Germany; 2 Cancer and Disease Epigenetics Research Group, Murdoch Childrens Research Institute, Royal Children’s Hospital, Flemington Road, Parkville, 3052 Victoria Australia; 3 Research Unit of Molecular Epidemiology, Institute of Epidemiology II, Helmholtz Zentrum Muenchen, Munich, Germany; 4 CHC St Vincent, Liège-Rocourt, Belgium; 5 Universitat Rovira I Virgili, Reus, Spain; 6 University of Milano, Milano, Italy; 7 Children’s Memorial Health Institute, Warsaw, Poland

## Abstract

Adiposity and obesity result from the interaction of genetic variation and environmental factors from very early in life, possibly mediated by epigenetic processes. Few Epigenome-Wide-Association-Studies have identified DNA-methylation (DNAm) signatures associated with BMI and body composition in children. Body composition by Bio-Impedance-Analysis and genome-wide DNAm in whole blood were assessed in 374 pre-school children from four European countries. Associations were tested by linear regression adjusted for sex, age, centre, education, 6 WBC-proportions according to Houseman and 30 principal components derived from control probes. Specific DNAm variants were identified to be associated with BMI (212), fat-mass (230), fat-free-mass (120), fat-mass-index (24) and fat-free-mass-index (15). Probes in genes *SNED1*(*IRE-BP1*), *KLHL6*, *WDR51A*(*POC1A*), *CYTH4-ELFN2*, *CFLAR*, *PRDM14*, *SOS1*, *ZNF643*(*ZFP69B*), *ST6GAL1*, *C3orf7*0*, CILP2*, *MLLT4* and ncRNA *LOC101929268* remained significantly associated after Bonferroni-correction of *P*-values. We provide novel evidence linking DNAm with (i) altered lipid and glucose metabolism, (ii) diabetes and (iii) body size and composition in children. Both common and specific epigenetic signatures among measures were also revealed. The causal direction with phenotypic measures and stability of DNAm variants throughout the life course remains unclear and longitudinal analysis in other populations is required. These findings give support for potential epigenetic programming of body composition and obesity.

## Introduction

There is mounting evidence that the risk of obesity extends far beyond simple energy imbalance arising from a combination of overeating and sedentary lifestyle. This is supported by the variable incidence of associated comorbidities in overweight and obese individuals (e.g. cardiovascular disease (CVD), type 2 diabetes (T2D) and non-alcoholic fatty liver disease (NAFLD)). A greater understanding of aetiology is imperative for prevention and appropriate intervention in both, children and adults^1–3^.

Conceptualising obesity as a metabolic disease, that is developmentally programmed and thus conditioned on developmental pathways and plasticity, substantially broadens the focus beyond the obvious areas of excess food intake, lack of physical activity and excess weight gain. This is key to unravelling the functional and programmed aetiologies of metabolic dysregulation that begin early in life in response to genetic and environmental (including nutritional) factors and are potentially mediated by epigenetic mechanisms over the life course^3–8^.

Epigenetic ‘plasticity’ is now widely thought to underpin many developmental processes in humans in response to a ‘perceived’ postnatal environment(s), with many observational studies in humans implicating this process in the programming of the developmental and metabolic pathways towards obesity^5,8–11^. Building on the key observations of Barker and others, there is now a considerable body of evidence that *in utero* and early life factors are critical in influencing subsequent metabolic risk, although the underlying mechanisms are largely unexplored. It is also clear that key metabolic risk factors, including body mass index (BMI), blood pressure and cholesterol, track from late childhood into adulthood^12,13^ (reviewed in^14–16^). However, the development of metabolic risk in the period between infancy and adolescence is poorly understood. Considerable successes have been reported in adults through Epigenome-wide Association Studies (EWAS), linking epigenetic variants (particularly DNAm) to specific metabolic phenotypes including obesity, BMI and T2D^17–23^. Some candidate gene studies have been carried out in children^24–27^. However, only few comprehensive EWAS analyses in childhood cohorts, with high quality body composition and metabolic measures, have been published specifically in early childhood^28–31^.

This study, part of the multicentre European Childhood Obesity Project (CHOP)^32–34^, aimed at investigating the association between measures of body size (absolute and WHO standardised BMI) and body composition (absolute and height standardised fat mass and fat free mass measures) with DNAm on a genome-wide scale in 374 pre-school children. Functional pathway analyses were used to elucidate the likely metabolic and biological functions of genes showing altered DNAm to build evidence of a plausible link with obesity and adiposity related traits.

## Results

### DNAm variation, BMI and WHO-Standardised BMI

A total of 212 differentially methylated probes (DMPs) located in or near 181 genes were significantly associated with BMI after accounting for multiple testing by FDR (Supplementary Table S1). The top ten are listed below (Table 1). Methylation levels at two probes (cg13850887, cg01706498), located in genes *SNED1* and *KLHL6*, remained significantly associated with BMI even after Bonferroni correction.

**Table 1:** Change in BMI, ZBMI, FM, FMI, FFM and FFMI for the top ten differentially methylated probes per percent change in DNA-methylation.

Rank	CpG ID	Gene	Change (SE) in BMI *	Rank	CpG ID	Gene	Change (SE) in ZBMI *
1	cg13850887	*SNED1*	−0.22 (0.04)^†‡^	1	cg13850887	*SNED1*	−0.12 (0.02)^†^
2	cg01706498	*KLHL6*	0.19 (0.03)^†‡^	2	cg01706498	*KLHL6*	0.11 (0.02)^†^
3	cg26401512	*ZNF643*	−0.20 (0.04)^†^	3	cg21126338	*FARP1*	0.20 (0.04)^†^
4	cg21525627	*(ZDHHC17)*	0.24 (0.05)^†^	4	cg21525627	*(ZDHHC17)*	0.15 (0.03)^†^
5	cg26867987	*COL11A2*	−0.18 (0.04)^†^	5	cg27285599	*TBCD;ZNF750*	0.21 (0.04)
6	cg17810765	*ANO7*	0.14 (0.03)^†^	6	cg17935297	*CILP2*	0.24 (0.05)
7	cg14518658	*(CYTH4-ELFN2)*	0.11 (0.02)^†^	7	cg24751284	*APEX1*	0.14 (0.03)
8	cg21126338	*FARP1*	0.32 (0.06)^†^	8	cg23416307	*GAK*	0.39 (0.08)
9	cg27285599	*TBCD;ZNF750*	0.34 (0.07)^†^	9	cg06369443	*KCNQ4*	0.20 (0.04)
10	cg17935297	*CILP2*	0.39 (0.08)^†^	10	cg26917480	*ADAP2*	0.15 (0.03)
**Rank**	**CpG ID**	**Gene**	**Change (SE) in FM** *	**Rank**	**CpG ID**	**Gene**	**Change (SE) in FMI** *
1	cg13850887	*SNED1*	−0.20 (0.03)^†‡^	1	cg13850887	*SNED1*	−0.15 (0.02)^†‡^
2	cg08692210	*WDR51A*	0.17 (0.03)^†‡^	2	cg04010122	*SOS1*	0.12 (0.02)^†‡^
3	cg14518658	*(CYTH4-ELFN2)*	0.10 (0.02)^†‡^	3	cg26627956	*CFLAR*	−0.14 (0.02)^†‡^
4	cg26627956	*CFLAR*	−0.20 (0.04)^†‡^	4	cg26401512	*ZNF643*	−0.12 (0.02)^†‡^
5	cg00384539	*PRDM14*	0.27 (0.05)^†‡^	5	cg15026574	*ST6GAL1*	−0.12 (0.02)^†‡^
6	cg04010122	*SOS1*	0.16 (0.03)^†^	6	cg14401837	*NPSR1*	−0.15 (0.03)^†^
7	cg04894009	*PRKDC*	0.32 (0.06)^†^	7	cg14518658	*(CYTH4-ELFN2)*	0.07 (0.01)^†^
8	cg13641993	*FBXO10*	0.23 (0.04)^†^	8	cg06594770	*TRIOBP*	0.24 (0.05)^†^
9	cg27038634	*(LOC101929268)*	0.09 (0.02)^†^	9	cg08692210	*WDR51A*	0.11 (0.02)^†^
10	cg15026574	*ST6GAL1*	−0.17 (0.03)^†^	10	cg04894009	*PRKDC*	0.21 (0.04)^†^
**Rank**	**CpG ID**	**Gene**	**Change (SE) in FFM** *	**Rank**	**CpG ID**	**Gene**	**Change (SE) in FFMI** *
1	cg27038634	*(LOC101929268)*	0.14 (0.02)^†‡^	1	cg17935297	*CILP2*	0.23 (0.04)^†‡^
2	cg08074767	*(MLLT4)*	−0.15 (0.03)^†‡^	2	cg06437396	*OSTM1*	0.12 (0.02)^†^
3	cg24332767	*C3orf70*	0.20 (0.04)^†‡^	3	cg26995653	*LINC01115 (TMEM18)*	0.07 (0.01)^†^
4	cg21126338	*FARP1*	0.38 (0.07)^†^	4	cg21525627	*(ZDHHC17)*	0.13 (0.03)^†^
5	cg07719679	*STEAP4*	0.18 (0.03)^†^	5	cg08074767	*(MLLT4)*	−0.07 (0.01)^†^
6	cg10135753	*BRSK1*	0.20 (0.04)^†^	6	cg25048701	*FOLR1*	0.26 (0.05)^†^
7	cg21525627	*(ZDHHC17)*	0.28 (0.05)^†^	7	cg13718870	*BRD3*	−0.07 (0.01)^†^
8	cg06376715	*TP73*	0.12 (0.02)^†^	8	cg15914340	*TSSC1*	0.30 (0.06)^†^
9	cg01577646	*RPS6KA2*	−0.21 (0.04)^†^	9	cg01706498	*KLHL6*	0.10 (0.02)^†^
10	cg25827873	*ERICH1*	−0.11 (0.02)^†^	10	cg19864468	*CHCHD5*	0.17 (0.03)^†^

*ß-coefficients from the EWAS regression analysis have been divided by 100 to scale these to a change in the respective outcome per percent change in the methylation ß-value (see method section). ^†^FDR significant (for uncorrected *P*-value and FDR *q*-value see Supplementary Tables S1 to S6). ^‡^Bonferroni significant (for corrected p-value see Supplementary Tables S1 to S6). Genes in brackets are those closest to probes of interest using the UCSC genome browser on human GRCh37/hg19 assembly.

Methylation level at only four sites (located in *SNED1, KLHL6, FARP1*, or 10kb upstream of *ZDHHC17*) was significantly associated (Table 1, Supplementary Table S2) with WHO standardised BMI (ZBMI) as an outcome. The top two (cg13850887, cg01706498) were consistent irrespective of BMI measure (Table 1), while variants in *FARP1*, *TBCD, ZNF750* and *CILP2* remained among the top ten significant probes in both comparisons.

**Figure 1:**
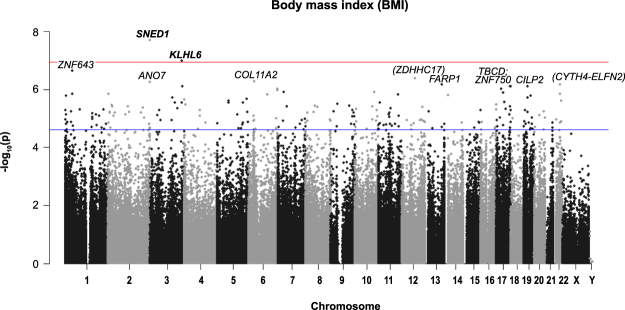
Manhattan plot of all HM450K probe *P*-values for the association of BMI (kg/m^2^) and methylation sites in preschool children. Top 10 sites are annotated. Red line indicates Bonferroni genome-wide significance and the blue line FDR significance. Chromosomal location of all HM450K probes is listed on the x-axis.

**Figure 2:**
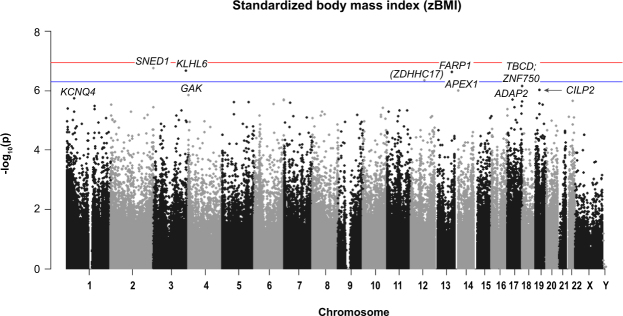
Manhattan plot of all HM450K probe *P*-values for the association of WHO-standardised BMI (z-score) and methylation sites in preschool children. Top 10 sites are annotated. Red line indicates Bonferroni genome-wide significance and the blue line FDR significance. Chromosomal location of all HM450K probes is listed on the x-axis.

A summary of *P*-values (Manhattan plot) for each probe association with BMI and WHO standardised BMI is provided in Figs 1 and 2.

### DNAm variation, FM and FMI

A total of 230 and 24 DMPs were significantly associated with absolute fat mass (FM, kg) and fat mass index (FMI, kg/m²), after FDR correction for multiple testing (Supplementary Tables S3, S4). These are associated with 199 and 22 gene regions respectively. Probes in *SNED1*, *WDR51A*, *CYTH4-ELFN2*, *CFLAR* and *PRDM14* remained significantly associated with FM, while those in *SNED1*, *SOS1*, *CFLAR*, *ZNF643* and *ST6GAL1* remained associated with FMI following Bonferroni correction (Table 1). The top DMP in both comparisons was cg13850887, located just downstream of a CpG island (South Shore) in the gene body of *SNED1* on chromosome 2.

**Figure 3:**
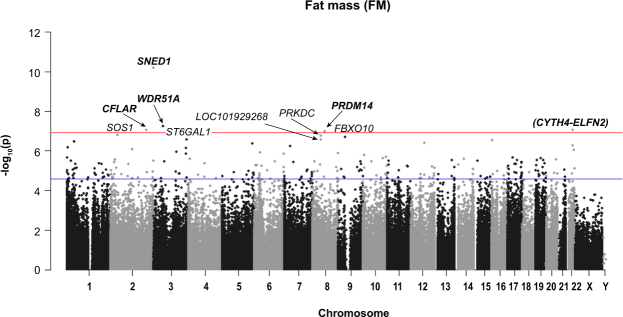
Manhattan plot of all HM450K probe *P*-values for the association of FM (kg) and methylation sites in preschool children. Top 10 sites are annotated. Red line indicates Bonferroni genome-wide significance and the blue line FDR significance. Chromosomal location of all HM450K probes is listed on the x-axis.

**Figure 4:**
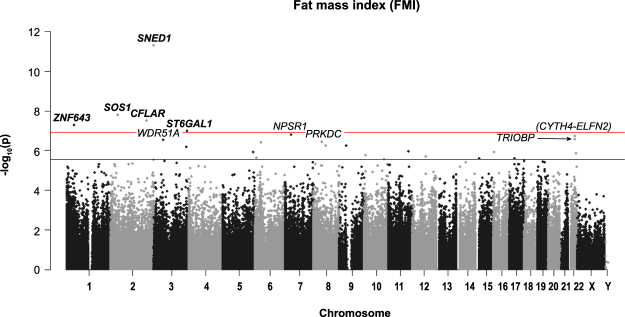
Manhattan plot of all HM450K probe *P*-values for the association of FMI (kg/m^2^) and methylation sites in preschool children. Top 10 sites are annotated. Red line indicates Bonferroni genome-wide significance and the blue line FDR significance. Chromosomal location of all HM450K probes is listed on the x-axis.

A summary of *P*-values (Manhattan plot) for each probe association with FM and FMI is provided in Figs 3 and 4).

### DNAm variation, FFM and FFMI

Absolute fat free mass (FFM, kg) and fat free mass index (FFMI, kg/m²) was significantly associated with 120 and 15 methylation variants (Supplementary Tables S5, S6), located in 109 and 14 gene regions, respectively. After Bonferroni correction for multiple testing, only three variants remained significantly associated with FFM, located in the non-coding RNA *LOC101929268*, upstream of *MLLT4* and in the uncharacterised *C3orf70* gene, while a single probe in gene *CILP2* was associated with FFMI (Table 1).

**Figure 5:**
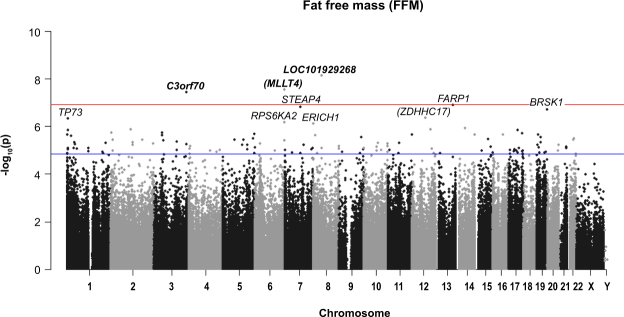
Manhattan plot of all HM450K probe *P*-values for the association of FFM (kg) and methylation sites in preschool children. Top 10 sites are annotated. Red line indicates Bonferroni genome-wide significance and the blue line FDR significance. Chromosomal location of all HM450K probes is listed on the x-axis.

**Figure 6:**
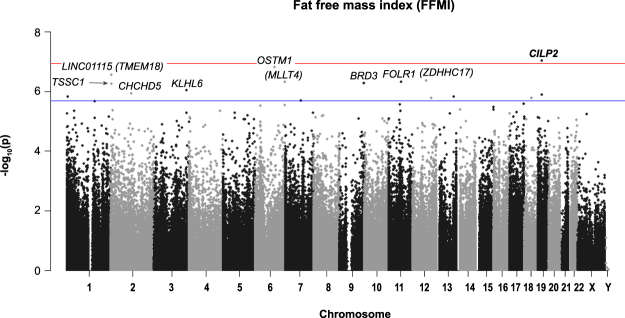
Manhattan plot of all HM450K probe *P*-values for the association of FFMI (kg/m^2^) and methylation sites in preschool children. Top 10 sites are annotated. Red line indicates Bonferroni genome-wide significance and the blue line FDR significance. Chromosomal location of all HM450K probes is listed on the x-axis.

A summary of *P*-values (Manhattan plot) for each probe association with FFM and FFMI is provided in Figs 5 and 6).

### Overlap of hits

**Figure 7:**
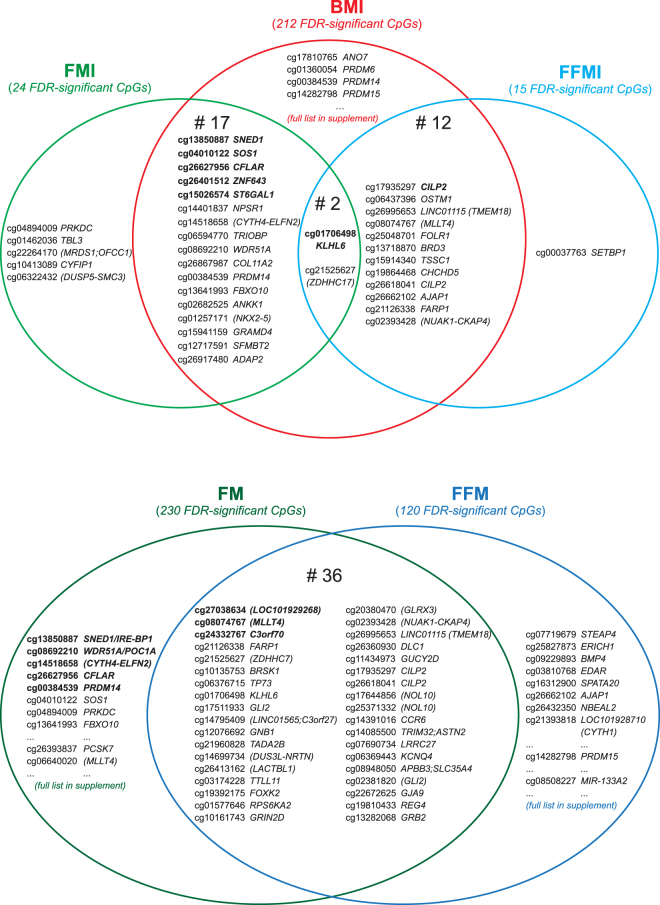
Overlap of differentially methylated probes (DMPs) associated with BMI, FMI and FFMI (**a**) and with FM and FFM (**b**) in preschool children. A full list of DMPs is provided in Supplementary Tables S1 to S6. Genes in brackets indicate manual annotation for closest genes using the UCSC genome browser on human GRCh37/hg19 assembly.

DMPs commonly associated with BMI, FMI and FFMI are presented in Fig. 7a. In total, two DMPs, one upstream of gene *ZDHHC17* (cg21525627) and another in *KLHL6* (cg01706498), were associated with all three measures with the same direction of effect. Of 24 DMPs associated with FMI, 19 were also associated with BMI. Out of 15 associated with FFMI, 14 were also associated with BMI.

Figure 7b shows the 36 DMPs associated with both absolute FM and FFM among the 230 and 120 hits, respectively. Among these, 24 are annotated to protein-coding gene bodies or their promoters. The remaining 12 are situated in gene-poor regions. One probe (cg26995653) is located in a long non-coding RNA *LINC01115* and the other (cg14795409) close to *LINC01565*. Despite some overlap in differential methylation, the majority of probes significantly associated with FM or FFM are specific for each measure.

### Functional Pathway Analysis

The resulting gene ontology (GO) terms for associations of DNAm with body size and composition measures are summarised in Supplementary Table S7. BMI-associations were over-represented for ‘Ras, MAPK, SHC-mediated signalling cascades by fibroblast growth factor receptors (FGFR1 to 4)’, and ‘Neurotrophin signalling pathways’. ‘Regulation of cytoskeleton organization’, ‘cardiac and striated muscle cell proliferation’ and ‘neuronal system’ were also enriched. Genes showing differential methylation in association with FM were over-represented for ‘Ras and Neurotrophin signalling pathways’, ‘spleen development’, ‘muscle cell proliferation’ and ‘pro-B cell differentiation’, while those associated with FFM included ‘Hippo signalling’, ‘negative regulation of growth’, ‘odontogenesis’, ‘cartilage development’ and ‘gliogenesis and neuron fate commitment’.

## Discussion

In this study of peripheral blood from 374 European pre-school children with highly accurate measures of body composition, we identified 212 differentially methylated probes (DMPs) associated with BMI, 230 DMPs with FM, 120 DMPs with FFM, 24 DMPs with FMI and 15 DMPs with FFMI. These probes measure DNAm in CpG sites that generally reside in protein-coding genes and non-coding RNAs previously linked with inflammation, glucose and lipid metabolism, browning of white fat, obesity and diabetes. Thus, many of the associations potentially make sense from a biological perspective; though only limited information is currently available in several instances (see below). Our finding that some DMPs are specifically associated with only one of the body size or composition measures BMI, FM, FMI, FFM, and FFMI whereas others are common to some or all measures e.g. FM and FFM, may be worthwhile investigating in more depth in future longitudinal studies to gain further insights into the process of body composition and obesity development and the role such potential epigenetic markers may play.

In the following paragraphs we provide some information on the 13 genes in which DNAm variants significantly associated after Bonferroni correction are located or are close by to add to the biological meaning and plausibility of our findings. These genes are *SNED1(IRE-BP1)*, *KLHL6*, *WDR51A(POC1A)*, *CYTH4-ELFN2*, *CFLAR*, *PRDM14*, *SOS1*, *ZNF643(ZFP69B), ST6GAL1, C3orf70*, *MLLT4, CILP2* and the ncRNA *LOC101929268*.

***SNED1*** (*Sushi, nidogen and EGF like domains*), also known as *IRE-BP1* (insulin-responsive sequence DNA-binding protein 1), activates insulin responsive genes *IGF-I*, *IGFBP-1* and *IGFBP-3* ^35^. In is expressed in insulin-responsive tissues such as fat and muscle^35^ and in hypothalamic regions involved in control of appetite and energy balance^36^. In animal models, overexpression of *SNED1* reduces^37^, but in some instances also increases^38^, hyperglycaemia and diabetes associated phenotypes. In our study, DMP cg13850887 at *SNED1* was inversely associated with BMI, FM and FMI after Bonferroni correction.

***KLHL6*** (*Kelch like family member 6*) regulates B cell differentiation and potentially plays a role in diabetes, as it appears up-regulated in islet cells of mice with type 1 diabetes (T1D)^39^, in children with pre-T1D^40^ and in post-mortem pancreatic tissue of adult T1D patients^41^. In our study, DMP cg01706498 within this gene was positively associated with BMI after Bonferroni correction and with ZBMI, FM, FMI, FFM and FFMI according to FDR.

***WDR51A*** (*WD repeat domain 51A*), *also known as POC1A (centriolar protein A)* have been found to show mutations in the centrosomal gene for individuals with primordial dwarfism and extreme insulin resistance^42^. We found a positive association between DNAm for DMP cg08692210 at this region with FM after Bonferroni correction and with BMI and FMI according to FDR.

***CYTH4-ELFN2*** gene region (*cytohesin 4, extracellular leucine-rich repeat and fibronectin type III domain containing 2*) was closest to the DMP cg14518658 associated with FM that we found in our study after Bonferroni correction and with BMI and FMI according to FDR. This region overlaps with binding sites of many transcription factors including the insulator protein CTCF and is close to a cluster of piwi-interacting RNAs (piRNAs) that have been linked to nutritional status and paternal inheritance of obesity^43,44^. Interestingly, expression of the two flanking genes is affected by diet: CYTH4, involved in vesicular secretion becomes upregulated in brown adipose tissue upon diet-induced obesity^45^ whereas ELFN2, a protein phosphatase regulator, becomes upregulated upon switch to Nordic diet^46^.

***CFLAR*** (*CASP8 and FADD like apoptosis regulator*), a gene involved in regulating the pro-apoptotic protein CFLAR, is upregulated in peripheral blood mononuclear cells of obese children and adolescents and normalises after BMI reduction^47^. We found an inverse association of DNAm for DMP cg26627956 within this gene with FM and FMI after Bonferroni correction and with BMI according to FDR.

***PRDM14*** (*PR/SET domain 14, previously PR domain containing 14*) is a pluripotency gene belonging to the PRDM family of transcriptional regulators. This family includes *PRDM16*, which promotes differentiation of adult skeletal muscle stem cells^48^ and PRDM4, which stimulates browning of white adipose tissue^49^. We found a positive association of DMP cg00384539 in the *PRDM14* gene with FM after Bonferroni correction and with BMI and FMI according to FDR. Interestingly, we found further associations within the PRDM family: DMP cg14282798 in *PRDM15* was associated with BMI and FFM and DMP cg01360054 in *PRDM6* with BMI according to FDR.

***SOS1*** (*SOS Ras/Rac guanine nucleotide exchange factor 1*) was recently reported to be transcriptionally upregulated in association with BMI in young adult monozygotic BMI-discordant Finnish twins^50^. A DMP (cg04010122) within this gene was identified in our study, which was associated, according to Bonferroni, with FMI and according to FDR with FM.

***ZNF643***(*Zinc finger protein 643 also known as ZFP69B*), a putative transcription factor gene, is situated next to *ZFP69*, which has been linked to pathogenesis of human diabetes, as its allelic variation associates with impaired lipid storage in white adipose tissue^51^. We found an inverse association of DNAm at cg26401512 in this gene with FMI according to Bonferroni correction and with FM according to FDR.

***ST6GAL1*** (*ST6 beta-galactosamide alpha-2,6-sialyltranferase 1*) codes for a membrane-bound glycosyltransferase and has been found to have a strong linkage with a high impact genetic variant in the adiponectin gene *ADIPOQ* that affects adiponectin plasma levels in Hispanics^52^. In our study DMP cg15026574 was inversely associated with FMI according to Bonferroni correction and with BMI and FM according to FDR.

***C3orf70*** (*Chromosome 3 open reading frame 70*). Gene polymorphisms in that gene code for an unknown protein and has also been found to have a strong linkage with a high impact genetic variant in adiponectin gene *ADIPOQ* that affects adiponectin plasma levels in Hispanics^52^. In our study, DMP cg 24332767 was positively associated with FFM after Bonferroni correction, and with BMI and FM according to FDR. Interestingly, *KLHL6* gene is also located around 3 Mb upstream of this region on chromosome 3q26-27.

***LOC101929268*** (*uncharacterised noncoding RNA*). This *ncRNA* gene was closest to the DMP cg27038634 that we found in our study to be associated with FFM after Bonferroni correction and with BMI and FM according to FDR. *LOC101929268* is upstream of the *EFCAB1* gene for which altered methylation was detected in adipose tissue of individuals with type 2 diabetes^53^.

**MLLT4** (myeloid/lymphoid or mixed-lineage leukaemia, translocated to, 4, also known as AFDN = afadin, adherens junction formation factor). This gene encodes a multi-domain protein involved in signalling and organisation of cell junctions during embryogenesis according to a pubmed/gene and HGNC search (http://www.genenames.org/) and was originally detected in relation to mouse development^54^. In our study, DMP cg08074767 near this gene was inversely associated with FFM after Bonferroni correction and with BMI, FM and FFMI according to FDR.

***CILP2*** (*Cartilage intermediate layer protein 2*). Genetic variation in *CILP2* antagonizes insulin-like growth factor (IGF-I) activity in chondrocytes^55^ and has been linked with serum lipid levels^56^ and type 2 diabetes^57^. Methylation at *CILP2* (cg17935297) was the top hit association for FFMI in our study after Bonferroni correction and was associated with BMI, FM and FFM according to FDR.

Two DNAm variants cg01706498 in *ZDHHC17* and cg21525627 in *KLHL6* were in our EWAS associated with all six (BMI, ZBMI, FM, FMI, FFM and FFMI) body size and composition measures after applying FDR threshold were found. *ZDHHC17* encodes for a palmitoyltransferase that regulates glucose homeostasis in adipocytes and has been proposed as a candidate gene for type 1 diabetes^58–60^.

It is also of interest that we found in this EWAS of the CHOP study, a methylation variant located in an unknown non-coding RNA *LINC01115* located approx. 100 kb downstream of the ‘obesity gene’ *TMEM18* that is expressed in the hypothalamus and has been linked with childhood obesity^61^, early onset extreme obesity^62^ and adult obesity^6^.

None of the identified differently methylated positions (DMP) in our study were found in respective EWAS in children and adolescence^28,29,31^. However, DMP in the same genes often just in some 1000 bp distance of each other were found in an EWAS conducted in West Australian obese 12 year old boys^28^. Associations with phenotypes at DMP in common genes include the genes *ANKRD11* (BMI, FM), *BAT2* (BMI, FM), *BCL11A* (FM), *C2orf85* (BMI), *C4orf22* (BMI), *CCNL2* (BMI), *CCR6* (BMI, FM, FFM), *CDH13* (BMI), *CNGA3* (BMI, FM), *CYFIP1* (FM, FMI), *DIABLO* (BMI), *DUSP5* (FMI), *FAM188B* (BMI), *FGFR2* (BMI), *FOXK2* (BMI, FM, FFM), *GNA12* (BMI), *HIPK2* (BMI), *IGF2R* (BMI), *INPP5A* (BMI, FFM), *JARID*2 (FM), *MACROD1* (BMI), *MAD1L1* (FFM), *MCF2L* (FM), *MEGF11* (BMI, FM), *MLLT4* (FM, FFM, FFMI), *MXD3* (FFM), *NPSR1* (BMI, FM, FMI), *PRKDC* (FM, FMI), *PTPRN2* (FFM), *RAB5C* (BMI, FM), *RERE* (FM), *RPS6KA2* (FM, FFM), *SFMBT2* (BMI, FM, FMI), *TBCD* (BMI, FM), *TNXB* (FM, FFM), *TRAPPC9* (FM), and *TRIM39* (BMI, FM). Supplementary Tables 8 to 12 list all FDR-significant DMPs of the CHOP study for the phenotypes separately and those DMPs of the Australian study that have at least a 5% difference in methylation among obese boys and an age-matched control group located within the above mentioned common genes^28^.

Several genes in our study have also been found differentially methylated in relation to BMI and obesity in previous EWAS in adults^18,21,63^.

An EWAS in an Arab population found a genome-wide significant inverse association of BMI with a DMP (cg17501210) in gene *RPS6KA2* (Chr6:166970252) in whole blood of adults^18^. We found in the same gene at DMP (cg01577646, Chr6:166911121) an inverse association with FM and FFM according to FDR criteria.

In an EWAS in African American adults, a genome-wide significant positive association with BMI was found at DMP (cg09664445, Chr17: 2612406) in gene *KIAA0664* ^21^. We also found a positive association with BMI in the same gene at DMP (cg09927637, Chr17: 2606848) according to FDR criteria.

In a very recent EWAS conducted in adult European and Indian Asian populations, a large number (187 markers) of associations of DNAm and BMI were identified and replicated in several populations and some in adipose tissue^63^. In our study, we found several DMP located in the same genes associated with BMI or other phenotypes. Associations with phenotypes at DMP in common genes include *ANKRD11*(BMI, FM), *JARID2* (FM), *MAD1L1* (FFM), *USP22* (BMI), *RPS6KA2* (FM, FFM), *SLC41A1* (FM), *SMC3* (FMI) and *ZC3H3* (BMI, FM, FFM). Supplementary Tables S13 to S16 list all overlaps in methylated genes of our study in European children and the EWAS in European or Indian Asian adults for the phenotypes separately^63^.

However, in contrast to several previous studies^21,22,26,29^, we did not detect differential methylation at *HIF3A* gene locus that has been detected in obese adults and children. In fact in our study, the smallest *uncorrected P*-value for an association of phenotype BMI, ZBMI, FM, FMI, FFM and FFMI with a methylation-site in gene *HIF3A* was *P* > 0.037, *P* > 0.048, *P* > 0.022, *P* > 0.018, *P* > 0.181 *and P* > 0.051 respectively for cg26749414 (cg02879662 for FFM). For all other methylation sites in *HIF3A* associations showed uncorrected *P*-values well above *P* > 0.11 (see Supplementary Tables S17 and S18). Nevertheless, we cannot rule out that this finding is due to a lack in power resulting from our sample size of 374 children, as some of the EWAS in adults are based on substantially larger samples.

This EWAS study has several strengths. It is one of the first to compare genome-wide DNAm among several body size and body composition measures in pre-school children with a reasonably large sample size. With the exception of one EWAS study^29^ in children that focused in their publication mainly on BMI - no EWAS in children investigated methylation in more than one body composition measure and in fat-mass or fat-free mass measured by bio-impedance analysis as in our study. This EWAS study provides novel evidence to the relatively less investigated field of epigenetics of childhood body composition. As demonstrated above, our findings are plausible from a biological point of view despite the necessarily explorative character intrinsic in EWAS analyses with a cross-sectional design. Our finding of specific and common methylation of body composition measures - if replicable in other populations – implies a promising potential for future research into the ways of epigenetic programming of obesity.

This EWAS study also has some limitations. First and foremost, a replication of our findings in further pre-school populations is required, despite the fact that some results were in line with previous studies in older children^28^ and in adult populations^18,21,63^. Although our sample size is larger than most of the few EWAS on body size or composition in children and adolescents^28,29,31^ a much larger sample size would be required to be sure that our null–association of DNAm variants within the *HIF3A* gene and BMI – shown previously in adults^21,22^ and children^26^ is not just a power problem. Moreover, we cannot rule out that other associated methylation sites, due to sample size, were missed.

A further limitation of our EWAS study is the cross-sectional design. Therefore we cannot determine the causal nature of our findings. In particular we cannot rule out that the found methylation levels are a consequence rather than a cause of our body composition phenotypes^63^. Moreover, the causal pathways may be even more complicated by genetic and other environmental influences, epigenetic mediation, modification and mechanisms for gene-environment interactions^64–66^. The list of corresponding methylation trait loci (mQTL) of our findings with those of the ARIES study, listed in Supplementary Tables S19 to S22, points in that direction^66^.

The issue of tissue specific methylation is a further limitation of our study – evaluating DNA-methylation in children’s blood and not in adipose tissue (for practical and ethical reasons) may have missed the true methylation markers associated with body composition^67,68^. In EWAS in adults it has been shown that DNAm assessed from subcutaneous adipose tissue was associated with BMI, DXA-measured centrally located fat and body fat distribution, whereas DNAm derived from blood was not^17^. Moreover, in a small EWAS of adult monozygotic twins discordant for obesity, it was shown that genome-wide DNAm variants determined from blood were not different among the twin-pairs. However, when stratifying the twin-pairs by level of liver fat accumulation, epigenetically different signatures were observed if the heavier co-twins had excessive liver fat^23^. On the other hand, this methods study reveals that if the metabolic status can be accounted for by metabolic measurements often associated with excess adipose tissue (higher levels of fasting glucose and insulin, higher-low-density lipoprotein, C-reactive protein, or higher diastolic blood pressure) differences in epigenetic profile relevant to the phenotype and tissue of interest may be detected from blood^23^.

A further issue is the high inflation according to genomic control lambda^69^ ranging from 1.24 to 146 for the 6 body size and composition measure models. High lambda values may be considered as casting some doubt on the number of methylation associations found. However, a recent methods study demonstrated that the usual inflation measure lambda is often an overestimate of the true inflation, as lambda is substantially increased dependent on the number of significant EWAS associations^70^. According to this recent method of “bacon” inflation, values in our study were substantially smaller, ranging from 1.10 to 1.17.

Using estimates of the 6 white blood cell types derived from adult blood according to the Houseman method^71^ and adjusting by this reference based approach for cell mix heterogeneity in pre-school children, could potentially be seen as a limitation of our analysis as blood cell mix levels from younger children differ somehow from those of adults. In fact, there is a vivid discussion on how to account for cell heterogeneity in EWAS^71–77^. However, adjustment with other reference based methods like Bakulski’s cord blood reference^72^ did not improve the genomic inflation lambda values in our study and most of the top 10 associations of the original analysis showed up as well. Using a reference based model to account for cell heterogeneity may be considered as a limitation in any case, as references derived from blood may not reflect the tissue-specificity of methylation of adipose tissue as discussed already above. However, using Houseman’s new reference free model “RefFreeEWAS”^74,75^ with K = 9 latent dimensions to account for potential collinearity of the phenotype and or covariates within the methylation value matrix, resulted in our study in an over-adjustment indicated by very low genomic inflation values (0.74 to 0.84) and extremely low “bacon”-inflation (0.10–0.21).

An implicit related limitation of our EWAS approach is that we cannot rule out that methylation values in our dataset are confounded due to collinearity with the body composition phenotypes. The reference free model potentially accounts for such collinearity. However, as mentioned above, applying the reference free method with the determined number of K = 9 estimated latent factors resulted in over-adjustment. Moreover, using just K = 8 or K = 7 latent adjustment factors resulted in quite different top 10 associations among the 3 models, despite the fact that genomic inflation lambdas were still below 1 even for K = 7. According to our knowledge it is still an open methodological research question to determine, which approach is best to use to account for cell heterogeneity in children’s EWAS – reference-based or reference-free methods. Therefore we have placed the results from the reference free approach in Supplementary Tables S23 to S28.

In the reported reference-based EWAS analyses maternal smoking during pregnancy was not adjusted for. This could be seen as a potential biasing factor. However, a repetition of the main EWAS analyses with this additional adjustment show that such a bias is limited. For all six body-size and composition measures almost all of the originally found top 10 associations with DNAm variants were also found within the top 10 in these additional adjusted analyses or were at least under the top 17. Moreover, these estimates did not differ substantially from those not adjusted for maternal smoking as documented in Supplementary Tables S29 to S34.

A further limitation of this EWAS is that no RNA data is available to study the associations of differential methylation at the found loci with gene expression. However, this may be worthwhile to investigate in further and larger EWAS studies.

In summary, we provide novel evidence linking DNAm at *SNED1(IRE-BP1*), *KLHL6*, *WDR51A*(*POC1A*), intergenic *CYTH4-ELFN2*, *CFLAR*, *PRDM14*, *SOS1*, *ZNF643*(*ZFP69B*), *ST6GAL1*, *C3orf70, MLLT4, CILP2* genes and noncoding RNA *LOC101929268* with altered lipid and glucose metabolism and differential body size and body composition measures in children. We also found methylation variation related to body composition in 39 genes previously found mostly in severely obese children but some also in adults. We also provide novel evidence about common and different epigenetic signatures between fat mass and fat free mass. The causal direction with phenotypic measures and stability of the DNAm variants throughout the life course remains unclear and longitudinal analysis in other populations is required. These findings give support for potential epigenetic programming of body composition and obesity and contribute to an emerging body of work linking specific exposures to variation in epigenetic profile and metabolic phenotypes in humans.

## Methods

### Study design and participants

This study is based on a subset of 374 children out of 543 children aged 5.5 years from study centres in Germany, Belgium, Italy and Spain of the European Childhood Obesity Trial Study (CHOP) registered at clinicaltrials.gov as NCT00338689 and URL: http://clinicaltrials.gov/ct2/show/NCT00338689?term=NCT00338689&rank=1. Details on the study have been published previously^32–34^. Inclusion criteria for this analysis were availability of blood buffy coats, valid exposure data on measured weight, height and Bio-Impedance Analysis (BIA) derived measurements of body composition (fat mass, fat free mass); all collected at 5.5 years of age in the children and information on basic characteristics of the offspring (sex, age of blood draw, country of study centre). Characteristics of the analysed study population are listed in Table 2.

**Table 2:** Characteristics of analysed study population.

	Boys	Girls	Total	*P*-value
	(n = 181)	(n = 193)	(n = 374)	Girls vs. Boys
Child’s birth weight (kg)	3.3 (0.3)	3.2 (0.3)	3.3 (0.3)	0.01
Length of gestation (weeks)	39.9 (1.2)	39.8 (1.2)	39.8 (1.2)	0.77
Child formula vs. Breastfed (n(%))	125 (69.1%)	121 (62.7%)	246 (65.8%)	0.20
Maternal age at delivery (years)	32.5 (4.2)	31.6 (4.1)	32.1 (4.1)	0.05
Maternal pre-pregnancy BMI (kg/m²)	23.9 (4.1)	23.2 (4.0)	23.5 (4.1)	0.15
Maternal smoking during pregnancy (n(%))	38 (21.0%)	37 (19.2%)	75 (20.1%)	0.66
Low parental education (n(%))	25 (13.8%)	12 (6.2%)	37 (9.9%)	0.02
Medium parental education (n(%))	95 (52.5%)	105 (54.4%)	200 (53.5%)	0.75
High parental education (n(%))	61 (33.7%)	76 (39.4%)	137 (36.6%)	0.24
Belgium (n(%))	23 (12.7%)	42 (21.8%)	65 (17.4%)	0.02
Germany (n(%))	12 (6.6%)	18 (9.3%)	30 (8.0%)	0.34
Italy (n(%))	79 (43.6%)	64 (33.2%)	143 (38.2%)	0.04
Spain (n(%))	67 (37.0%)	69 (35.8%)	136 (36.4%)	0.80
Child’s age at blood draw (months)	66.3 (0.9)	66.5 (0.8)	66.4 (0.8)	0.05
BMI (Kg/m²)	16.0 (2.1)	15.9 (1.6)	15.9 (1.9)	0.42
BMI (WHO-zscore)	0.4 (1.3)	0.3 (0.9)	0.3 (1.1)	0.33
Fat mass (kg)	4.3 (1.7)	4.6 (1.3)	4.5 (1.5)	0.02
Fat mass index (kg/m²)	3.3 (1.2)	3.6 (0.9)	3.4 (1.0)	0.01
Fat free mass (kg)	16.5 (2.0)	15.9 (2.0)	16.2 (2.1)	0.01
Fat free mass index (kg/m²)	12.7 (1.1)	12.3 (0.9)	12.5 (1.0)	<0.01

Data in table are mean (SD), n (%).

### Ethics statement

The CHOP study was conducted according to the principles expressed in the Declaration of Helsinki. The local ethics committees of each study centre approved all study procedures: Belgium (Comitè d’Ethique de L’Hopital Universitaire des Enfants Reine Fabiola; no. CEH 14/02), Germany (Bayerische Landesärztekammer Ethik-Kommission; no. 02070), Italy (Azienda Ospedaliera San Paolo Comitato Etico; no. 14/2002), Poland (Instytut Pomnik–Centrum Zdrowia Dziecka Komitet Etyczny; no 243/KE/2001), and Spain (Comité ético de investigación clinica del Hospital Universitario de Tarragona Joan XXIII). Written informed parental consent was obtained for each infant.

### Procedures

Weight and height was measured to the nearest 100 g and 0.1 cm during physical exam at age 5.5 years by a trained study personal according to strict SOPs using SECA 702 electronic scales (SECA, Hamburg; Germany) and SECA 242 stadiometer (SECA, Hamburg; Germany). BMI was calculated from these anthropometric measurements as child’s weight in kg divided by height in metres squared (BMI = (weight (kg)/height (m²)). BMI was also standardised according to WHO age- and sex–specific child growth standards (ZBMI) using a macro downloaded from http://www.who.int/childgrowth/en. Fat Mass in kg (FM) and Fat Free Mass in kg (FFM) were determined by the built-in equation of the Bio-Impedance-Analysis device (Tanita BC-418MA, Segmental Body Composition Analyser, Tanita Europe, Sindelfingen Germany). Fat mass index and fat free mass index were derived from these measures: FMI = FM (kg)/Height (m^2^) and FFMI = FFM (kg)/Height (m^2^).

During the child’s physical examination at age 5.5 years, blood was drawn to collect peripheral blood cells from buffy coats. These were used to determine DNA methylation (DNAm) status at a genome-wide level by using the Infinium HumanMethylation450 BeadChip (HM450K). DNA extraction, bisulfite conversion and methylation analysis were performed at the Genome Analysis Center of Helmholtz Zentrum Muenchen, Munich, Germany. Details were described previously^34^. In brief, genomic DNA was extracted using a standard precipitation procedure. Bisulfite conversion was performed using the EZ-96 DNA Methylation Kit (Zymo Research, Irvine, Ca; USA) and converted DNA samples were hybridised on the Infinium HumanMethylation450 BeadChip (HM450K) according to the manufactures instructions (Illumina Inc., San Diego, USA). Data pre-processing and normalisation were performed by the first author according to the approach of Touleimat and Tost^78^ with some adaptions e.g. the beta-mixture quantile normalization (BMIQ) step of the ß-values^79^ and exclusion of cross-reactive probes^80^ as described in detail previously^34^.

The final data set comprised DNA-methylation values (DNAm) at 431313 CpG sites in each of the 374 children for EWAS analysis.

### Statistical Analysis (EWAS)

Potential associations between each of the measures of body size (BMI, ZBMI) or body composition (FM, FMI, FFM, FFMI) and quality controlled but untransformed methylation values at each of 431313 CpG sites were determined by standard linear regression models with adjustment for child’s sex, age at blood draw (months), study centre (Germany (DE), Italy (IT), Spain(ES), reference Belgium (BE)), parental education (High = both parents 12 + yrs. of schooling, reference Middle = both parents 10 − <12 yrs. of schooling or only one parent 12 + years of schooling and Low = both parents achieved basic schooling only), the estimated proportions of six major white blood cell types (WBC), CD4 + T cells, CD8 + T cells, B cells, NK cells, monocytes and granulocytes according to Houseman’s method and the top 30 principal components (PC) derived from control probes on the HM450K platform^71,81^. All statistical analyses were performed with R-software version R3.3.2 of the R-project (https://www.r-project.org/) at the mainframe of the Leibniz-Rechenzentrum (LRZ) in Garching/Munich.

An association of a differently methylated CpG site (beta-values) with the respective body size or composition measure was considered significant at the epigenome-wide level, if the false discovery rate (FDR) was *q* < 0.05^82^. The estimates listed in tables in the result section or the appendix are the adjusted regression coefficient of the model described above where a CpG site enters the equation as methylation fraction (range 0 to 1) and is scaled to percent methylation by dividing the CpG related regression coefficient by 100. Therefore the shown estimates can be interpreted as the respective change in the analysed outcome for a one percent change in methylation of the respective CpG (predictor). Inflation of *P*-values for the assessed associations in this EWAS were computed according to both the usual genomic control inflation factor lambda^69^ and according to the recent “bacon” method that account for the number of significant associations^70^. Both inflation estimates are depicted in Q-Q-plots for each of the models of the body size and composition measures (see Supplementary Fig. S1).

### Functional characterization of differentially methylated CpGs

The identified CpG sites were annotated according to Illumina (www.illumina.com, HumanMethylation450_15017482_v1.csv) or manually using the UCSC genome browser, GRCh37/h19 assembly for non-annotated CpGs (https://genome.ucsc.edu). Ontology analyses were conducted using a fixed set gene enrichment analysis approach performed with g:Profiler (http://biit.cs.ut.ee/gprofiler/index.cgi)^83^. Pathway analysis included Gene Ontology (Biological Process, Cellular Component and Molecular Function), KEGG and Reactome gene-set databases. The analysis was performed on ranked gene lists (ranked according to *P*-value from EWAS regression analysis) with advanced options ‘Size of functional category’: 3 (min) to 500 (max) and ‘Size of Q&T’: min of 2 using the gSCS threshold that is more stringent than FDR.

### Data availability

Results are extensively documented in the supplement. To protect patient confidentiality, data is available upon request. Future interested researchers can make requests to Prof Dr Berthold Koletzko, email: office.koletzko@med.lmu.de

## Electronic supplementary material

Supplementary Info

Supplementary Table S1

Supplementary Table S2

Supplementary Table S3

Supplementary Table S4

Supplementary Table S5

Supplementary Table S6
