# Supplementary material for: DNA-Methylation and Body Composition in Preschool Children: Epigenome-Wide-Analysis in the European Childhood Obesity Project (CHOP)-Study
Source: Sci Rep. 2017 Oct 30;7:14349. doi: 10.1038/s41598-017-13099-4 (PMC5662763; doi:10.1038/s41598-017-13099-4)
Supplement: Supplementary file 1 — Supplementary Info [file 41598_2017_13099_MOESM1_ESM.pdf]

DNA-Methylation and Body Composition in Children: Epigenome-Wide-Analysis in the European Childhood Obesity Project (CHOP)-Study

Peter Rzehak, Marcela Covic, Richard Saffery, Eva Reischl, Simone Wahl, Veit Grote, Martina Weber, Annick Xhonneux, Jean-Paul Langhendries, Natalia Ferre, Ricardo Closa-Monasterolo, Joaquin Escribano, Elvira Verduci, Enrica Riva, Piotr Socha, Dariusz Gruszfeld and Berthold Koletzko

Supplementary Table S7: Gene ontology terms.[1]

	Pathway ID	Pathway description	Corrected *P*-value	Gene names
**BMI**	GO:0007265	Ras protein signal transduction	1.23E-02	FGFR2,CYTH4,FGF9,GRIN2D,SOS1,ARHGEF2,DUSP5, CDH13,DOK3,GNA12,FARP1,SPTBN4,DLC1,GRB2,NTRK1
	GO:0051493	regulation of cytoskeleton organization	1.73E-02	ABL1,TRIOBP,SMC3,ARHGEF2,TBCD,SNCA, HAUS6,SPTBN4,DLC1,GRB2,KIRREL
	GO:0051494	negative regulation of cytoskeleton organization	1.40E-02	TRIOBP,SMC3,ARHGEF2,TBCD,SNCA,HAUS6,SPTBN4,DLC1
	GO:0060043	regulation of cardiac muscle cell proliferation	4.03E-02	FGFR2,TP73,FGF9,NKX2-5
	GO:0014855	striated muscle cell proliferation	1.98E-02	CFLAR,FGFR2,TP73,FGF9,NKX2-5
	GO:0051258	protein polymerization	3.51E-02	ABL1,TRIOBP,TBCD,SNCA,SPTBN4,GRB2,KIRREL
	GO:0032271	regulation of protein polymerization	7.20E-03	ABL1,TRIOBP,TBCD,SNCA,SPTBN4,GRB2,KIRREL
	GO:0032272	negative regulation of protein polymerization	4.75E-02	TRIOBP,TBCD,SNCA,SPTBN4
	KEGG:04010	MAPK signaling pathway	1.79E-04	FGFR2,FGF9,SOS1,ECSIT,DUSP5,MAPK8IP3, GNA12,PTPRR,GRB2,CACNA1E,NTRK1
	KEGG:04722	Neurotrophin signaling pathway	4.24E-03	TP73,ABL1,SOS1,GRB2,NTRK1
	KEGG:05200	Pathways in cancer	1.15E-02	FGFR2,GLI2,ABL1,FGF9,SOS1,CTBP2,GRB2,NTRK1
	KEGG:05220	Chronic myeloid leukemia	5.34E-03	ABL1,SOS1,CTBP2,GRB2
	KEGG:04014	Ras signaling pathway	2.20E-03	FGFR2,ABL1,FGF9,RAB5C,SOS1,MLLT4,GRB2
	REAC:5655332	Signaling by FGFR3 in disease	3.01E-02	FGF9,SOS1,GRB2
	REAC:8853338	Signaling by FGFR3 point mutants in cancer	3.01E-02	FGF9,SOS1,GRB2
	REAC:5654699	SHC-mediated cascade:FGFR2	4.24E-03	FGFR2,FGF9,SOS1,GRB2
	REAC:5654704	SHC-mediated cascade:FGFR3	1.61E-02	FGF9,SOS1,GRB2
	REAC:5654688	SHC-mediated cascade:FGFR1	2.60E-02	FGF9,SOS1,GRB2
	REAC:5654719	SHC-mediated cascade:FGFR4	3.45E-02	FGF9,SOS1,GRB2
	REAC:5655253	Signaling by FGFR2 in disease	3.26E-02	FGFR2,FGF9,SOS1,GRB2
**FM**	GO:0033002	muscle cell proliferation	1.60E-02	CFLAR,TP73,NKX2-5,PRKDC
	GO:0014855	striated muscle cell proliferation	2.54E-02	CFLAR,TP73,NKX2-5
	GO:0048536	spleen development	2.78E-02	JARID2,ABL1,CACNB4,NKX2-5,PRKDC
	GO:0002320	lymphoid progenitor cell differentiation	3.35E-02	SOS1,PRKDC
	GO:0002328	pro-B cell differentiation	9.87E-03	SOS1,PRKDC
	KEGG:04722	Neurotrophin signaling pathway	2.98E-02	RPS6KA2,TP73,ABL1,SOS1,NTRK1
	KEGG:04014	Ras signaling pathway	1.52E-02	GNB1,ABL1,FGF9,RAB5C,ZAP70,SOS1,MLLT4,GNG4,GRB2
	REAC:112308	Depolarization of the Presynaptic Terminal Triggers the Opening of Calcium Channels	3.97E-02	CACNB1,CACNB4,CACNA1E
	REAC:112316	Neuronal System	1.53E-03	CACNB1,RPS6KA2,GNB1,GRIN2D,KCNQ4, LRFN1,PTPRF,KCNH1,GNG4,KCNJ10,CACNB4,CACNA1E
**FMI**	GO:0002328	pro-B cell differentiation	2.49E-02	SOS1,PRKDC
**FFM**	GO:0061037	negative regulation of cartilage development	2.71E-02	GLI2,TGFB2,BMP4
	GO:0014013	regulation of gliogenesis	1.33E-02	TP73,BMP4,MLLT4
	GO:0042476	odontogenesis	2.32E-02	GLI2,TGFB2,GLI3,BMP4,EDAR,MSX1
	GO:0021520	spinal cord motor neuron cell fate specification	4.04E-02	GLI2,GLI3,ISL2
	GO:0048663	neuron fate commitment	1.60E-02	GLI2,TGFB2,BMP4,ISL2
	GO:0045926	negative regulation of growth	4.28E-02	TP73,TGFB2,TLL2,STC2,BMP4,SQSTM1,MSX1
	KEGG:04390	Hippo signaling pathway	1.64E-02	GLI2,TP73,TGFB2,BMP4
**FFMI**	GO:0035303	regulation of dephosphorylation	4.43E-02	NUAK1,FARP1
	GO:0035304	regulation of protein dephosphorylation	4.10E-02	NUAK1,FARP1
	GO:0010921	regulation of phosphatase activity	3.03E-02	NUAK1,FARP1
	GO:0045177	apical part of cell	9.13E-03	FOLR1,MLLT4,AJAP1

Supplementary Table S8: Methylation variants within the same genes in pre-school children of the CHOP study (BMI) and older children in the study of Huang. [2]

EWAS-BMI	CHOP						Huang et.al. 2015[2]						
Gene	Rank # of 212 FDR sign.	CpG ID	CHR	Position (bp)	Change (SE) in BMI per % change in DNAm	uncorrected *P*-value	Rank # of 1879 with 5% DNAm diffrence	CpG ID	CHR	Position * (bp)	% DNAm control	% DNAm obese	uncorrected *P*-value
ANKRD11	40	cg26666886	16	89558024	-0.32 (0.07)	3.37E-06	1715	cg06159562	16	89424790	0.4192	0.5062	0.0434
	59	cg27267258	16	89359175	0.35 (0.07)	5.06E-06	301	cg27660627	16	89461800	0.5852	0.5303	0.0048
BAT2	124	cg22503047	6	31601411	-0.30 (0.07)	1.20E-05	1632	cg18685724	6	31587910	0.4091	0.4914	0.0403
C2orf85	195	cg09034753	2	242810748	0.09 (0.02)	2.22E-05	869	cg11654325	2	243000000	0.611	0.6644	0.017
							728	cg23491841	2	243000000	0.5682	0.6371	0.0139
C4orf22	202	cg02256198	4	81306753	0.21 (0.05)	2.30E-05	1554	cg23548438	4	81257820	0.542	0.4908	0.038
CCNL2	114	cg04880874	1	1322461	0.26 (0.06)	1.12E-05	1871	cg24837149	1	1333778	0.3151	0.2411	0.0495
							196	cg24849049	1	156000000	0.6748	0.6189	0.0031
CCR6	15	cg14391016	6	167523797	0.24 (0.05)	1.06E-06	1055	cg13615963	6	168000000	0.1975	0.1446	0.0216
CDH13	197	cg16777782	16	82671333	-0.24 (0.06)	2.23E-05	1869	cg09044981	16	82827680	0.8276	0.7591	0.0494
CNGA3	84	cg25734624	2	99013247	0.19 (0.04)	7.28E-06	1182	cg11959694	2	98962560	0.5306	0.5822	0.0252
DIABLO	127	cg10802680	12	122712075	0.15 (0.03)	1.23E-05	704	cg19529732	12	123000000	0.5232	0.5773	0.0133
							106	cg21805118	12	123000000	0.6445	0.7013	0.0016
FAM188B	123	cg07831312	7	30855293	-0.16 (0.04)	1.20E-05	1004	cg07136111	7	30829000	0.4717	0.4098	0.0201
FGFR2	128	cg14834653	10	123353791	-0.18 (0.04)	1.24E-05	1472	cg02210151	10	123000000	0.5832	0.6616	0.0349
FOXK2	47	cg05945266	17	80545020	0.24 (0.05)	3.89E-06	907	cg02116768	17	80545320	0.4588	0.363	0.018
GNA12	179	cg24824703	7	2801248	0.19 (0.04)	1.97E-05	215	cg16569650	7	2773072	0.6423	0.5858	0.0035
HIPK2	117	cg03520802	7	139273697	0.12 (0.03)	1.15E-05	1121	cg15039797	7	139000000	0.8082	0.8597	0.0232
IGF2R	191	cg19196862	6	160491078	-0.25 (0.06)	2.17E-05	224	cg09555124	6	160000000	0.7605	0.8191	0.0036
INPP5A	199	cg08552167	10	134560109	-0.28 (0.07)	2.29E-05	1848	cg01040749	10	134000000	0.7154	0.6511	0.0485
							487	cg09163720	10	134000000	0.66	0.5872	0.0086
MACROD1	100	cg20219100	11	63776397	0.26 (0.06)	8.84E-06	659	cg10675058	11	63858880	0.6102	0.5397	0.0122
MEGF11	190	cg11841546	15	66195556	-0.26 (0.06)	2.14E-05	835	cg00682263	15	66188800	0.5975	0.4801	0.0163
NPSR1	17	cg14401837	7	34697493	-0.24 (0.05)	1.26E-06	1183	cg17220237	7	34911380	0.8167	0.758	0.0252
RAB5C	18	cg17989572	17	40306700	-0.50 (0.10)	1.32E-06	338	cg00435173	17	40284050	0.5646	0.5127	0.0054
SFMBT2	56	cg12717591	10	7205138	0.42 (0.09)	4.74E-06	209	cg05103064	10	7311500	0.7742	0.8312	0.0034
	130	cg19104196	10	7214172	0.25 (0.06)	1.28E-05	209	cg05103064	10	7311500	0.7742	0.8312	0.0034
TBCD	139	cg18699524	17	80765560	0.24 (0.05)	1.37E-05	1866	cg22175624	17	80829260	0.7176	0.7735	0.0493
	9	cg27285599	17	80798023	0.34 (0.07)	7.76E-07	1866	cg22175624	17	80829260	0.7176	0.7735	0.0493
TRIM39	94	cg01363869	6	30309848	-0.17 (0.04)	8.28E-06	1482	cg03094134	6	30297170	0.4733	0.4204	0.0353
							1633	cg21426759	6	30303130	0.6424	0.5747	0.0403

* Positions as listed in Huang 2015, Supplementary Table 2..[2]

Supplementary Table S9: Methylation variants within the same genes in pre-school children of the CHOP study (FM) and older children in the study of Huang. [2]

EWAS-FM	CHOP						Huang et al. 2015						
Gene	Rank # of 230 FDR sign.	CpG ID	CHR	Position (bp)	Change (SE) in FM per % change in DNAm	uncorrected *P*-value	Rank # of 1879 with 5% DNAm diffrence	CpG ID	CHR	Position * (bp)	% DNAm control	% DNAm obese	uncorrected *P*-value
ANKRD11	84	cg26666886	16	89558024	-0.25 (0.05)	7.43E-06	1715	cg06159562	16	89424790	0.4192	0.5062	0.0434
							301	cg27660627	16	89461800	0.5852	0.5303	0.0048
BAT2	135	cg22503047	6	31601411	-0.25 (0.06)	1.38E-05	1632	cg18685724	6	31587910	0.4091	0.4914	0.0403
BCL11A	152	cg23718924	2	60778617	-0.30 (0.07)	1.57E-05	1699	cg16951074	2	60755360	0.6608	0.6052	0.0428
							1074	cg24361098	2	60748950	0.5167	0.5881	0.0219
CCR6	110	cg14391016	6	167523797	0.17 (0.04)	1.10E-05	1055	cg13615963	6	168000000	0.1975	0.1446	0.0216
CNGA3	62	cg25734624	2	99013247	0.16 (0.03)	4.77E-06	1182	cg11959694	2	98962560	0.5306	0.5822	0.0252
CYFIP1	43	cg10413089	15	22921426	0.20 (0.04)	3.24E-06	1377	cg11403739	15	22923740	0.3569	0.4211	0.0315
							922	cg12759523	15	22923500	0.6017	0.6879	0.0184
							282	cg12792931	14	77499160	0.4932	0.5492	0.0046
							272	cg17896808	15	22992840	0.7177	0.7723	0.0044
FOXK2	157	cg05945266	17	80545020	0.18 (0.04)	1.60E-05	907	cg02116768	17	80545320	0.4588	0.363	0.018
FOXK2	50	cg19392175	17	80554772	0.15 (0.03)	3.65E-06	1867	cg02141929	16	56681540	0.6849	0.7449	0.0493
JARID2	68	cg14388804	6	15521099	0.25 (0.05)	5.54E-06	1640	cg18188328	6	15505090	0.6423	0.5853	0.0407
MCF2L	229	cg22699527	13	113714917	-0.52 (0.12)	2.64E-05	1133	cg05669497	13	114000000	0.2208	0.1561	0.0237
MEGF11	230	cg11841546	15	66195556	-0.20 (0.05)	2.66E-05	835	cg00682263	15	66188800	0.5975	0.4801	0.0163
MLLT4	78	cg06640020	6	168352700	-0.32 (0.07)	6.93E-06	362	cg00686132	6	168000000	0.6269	0.545	0.0058
NPSR1	16	cg14401837	7	34697493	-0.21 (0.04)	5.74E-07	1183	cg17220237	7	34911380	0.8167	0.758	0.0252
PRKDC	7	cg04894009	8	48695125	0.32 (0.06)	1.78E-07	1309	cg11773920	8	48686400	0.678	0.7307	0.0292
PTPRN2	116	cg24093300	7	157543805	-0.14 (0.03)	1.20E-05	179	cg00280858	7	158000000	0.7191	0.6655	0.0028
							245	cg11111139	7	158000000	0.5867	0.5297	0.004
							697	cg13736939	7	158000000	0.5262	0.4651	0.013
RAB5C	59	cg17989572	17	40306700	-0.38 (0.08)	4.60E-06	338	cg00435173	17	40284050	0.5646	0.5127	0.0054
RERE	224	cg26915618	1	8427379	0.25 (0.06)	2.54E-05	395	cg23963229	1	8725074	0.8811	0.9472	0.0065
RPS6KA2	120	cg01577646	6	166911121	-0.13 (0.03)	1.26E-05	1541	cg08557970	6	167000000	0.4913	0.438	0.0375
SFMBT2	26	cg12717591	10	7205138	0.36 (0.07)	1.62E-06	209	cg05103064	10	7311500	0.7742	0.8312	0.0034
	81	cg19104196	10	7214172	0.21 (0.05)	7.14E-06	209	cg05103064	10	7311500	0.7742	0.8312	0.0034
TBCD	128	cg27285599	17	80798023	0.24 (0.05)	1.34E-05	1866	cg22175624	17	80829260	0.7176	0.7735	0.0493
TNXB	204	cg25270367	6	32022898	-0.02 (0.00)	2.28E-05	1673	cg00872984	6	32063990	0.4172	0.4836	0.0421
							1280	cg07524919	6	32063900	0.4017	0.49	0.0284
							548	cg10365886	6	32063870	0.5771	0.6565	0.0098
							137	cg14188106	6	32063900	0.4143	0.5163	0.0021
TRAPPC9	212	cg24841559	8	141034008	0.19 (0.04)	2.36E-05	803	cg16191297	8	141000000	0.7429	0.8567	0.0156
TRIM39	169	cg01363869	6	30309848	-0.13 (0.03)	1.78E-05	1482	cg03094134	6	30297170	0.4733	0.4204	0.0353
							1633	cg21426759	6	30303130	0.6424	0.5747	0.0403

* Positions as listed in Huang 2015, Supplementary Table 2..[2]

Supplementary Table S10: Methylation variants within the same genes in pre-school children of the CHOP study (FMI) and older children in the study of Huang. [2]

EWAS-FMI	CHOP						Huang et. al. 2015						
Gene	Rank # of 24 FDR sign.	CpG ID	CHR	Position (bp)	Change (SE) in FM per % change in DNAm	uncorrected *P*-value	Rank # of 1879 with 5% DNAm diffrence	CpG ID	CHR	Position * (bp)	% DNAm control	% DNAm obese	uncorrected *P*-value
CYFIP1	23	cg10413089	15	22921426	0.13 (0.03)	2.51E-06	1377	cg11403739	15	22923740	0.3569	0.4211	0.0315
							922	cg12759523	15	22923500	0.6017	0.6879	0.0184
							272	cg17896808	15	22992840	0.7177	0.7723	0.0044
DUSP5	24	cg06322432	10	112289853	0.07 (0.01)	2.70E-06	1043	cg18756771	10	112000000	0.7675	0.7108	0.0212
NPSR1	6	cg14401837	7	34697493	-0.15 (0.03)	1.59E-07	1183	cg17220237	7	34911380	0.8167	0.758	0.0252
PRKDC	10	cg04894009	8	48695125	0.21 (0.04)	3.60E-07	1309	cg11773920	8	48686400	0.678	0.7307	0.0292
SFMBT2	19	cg12717591	10	7205138	0.24 (0.05)	1.78E-06	209	cg05103064	10	7311500	0.7742	0.8312	0.0034

* Positions as listed in Huang 2015, Supplementary Table 2..[2]

Supplementary Table S11: Methylation variants within the same genes in pre-school children of the CHOP study (FFM) and older children in the study of Huang. [2]

EWAS-FFM	CHOP						Huang et al. 2015						
Gene	Rank # of 120 FDR sign.	CpG ID	CHR	Position (bp)	Change (SE) in FM per % change in DNAm	uncorrected *P*-value	Rank # of 1879 with 5% DNAm diffrence	CpG ID	CHR	Position * (bp)	% DNAm control	% DNAm obese	uncorrected *P*-value
CCR6	38	cg14391016	6	167523797	0.25 (0.05)	3.81E-06	1055	cg13615963	6	168000000	0.1975	0.1446	0.0216
FOXK2	92	cg19392175	17	80554772	0.19 (0.04)	1.03E-05	907	cg02116768	17	80545320	0.4588	0.363	0.018
INPP5A	59	cg09602753	10	134574192	-0.31 (0.07)	6.74E-06	1848	cg01040749	10	134000000	0.7154	0.6511	0.0485
							487	cg09163720	10	134000000	0.66	0.5872	0.0086
MAD1L1	85	cg05985070	7	2050170	-0.20 (0.04)	9.59E-06	1714	cg05095590	7	2139259	0.5284	0.5866	0.0434
							1349	cg18650626	7	1914073	0.8267	0.8891	0.0308
MLLT4	2	cg08074767	6	168113741	-0.15 (0.03)	2.91E-08	362	cg00686132	6	168000000	0.6269	0.545	0.0058
MXD3	114	cg04586126	5	176734633	0.15 (0.03)	1.33E-05	1768	cg25213720	5	177000000	0.4232	0.3494	0.0455
PTPRN2	66	cg26075417	7	157544211	0.57 (0.13)	7.86E-06	179	cg00280858	7	158000000	0.7191	0.6655	0.0028
							245	cg11111139	7	158000000	0.5867	0.5297	0.004
							697	cg13736939	7	158000000	0.5262	0.4651	0.013
RPS6KA2	9	cg01577646	6	166911121	-0.21 (0.04)	6.85E-07	1541	cg08557970	6	167000000	0.4913	0.438	0.0375
TNXB	48	cg04147642	6	32054659	0.19 (0.04)	5.22E-06	1673	cg00872984	6	32063990	0.4172	0.4836	0.0421
							548	cg10365886	6	32063870	0.5771	0.6565	0.0098
							137	cg14188106	6	32063900	0.4143	0.5163	0.0021

* Positions as listed in Huang 2015, Supplementary Table 2..[2]

Supplementary Table S12: Methylation variants within the same genes in pre-school children of the CHOP study (FFMI) and older children in the study of Huang. [2]

EWAS-FFMI	CHOP						Huang et al. 2015						
**Gene**	**Rank # of 15 FDR sign.**	**CpG ID**	**CHR**	**Position (bp)**	**Change (SE) in FM per % change in DNAm**	**uncorrected *P*-value**	**Rank # of 1879 with 5% DNAm diffrence**	**CpG ID**	**CHR**	**Position * (bp)**	**% DNAm control**	**% DNAm obese**	**uncorrected *P*-value**
MLLT4	5	cg08074767	6	168113741	-0.07 (0.01)	4.71E-07	362	cg00686132	6	168000000	0.6269	0.545	0.0058

* Positions as listed in Huang 2015, Supplementary Table 2..[2]

Supplementary Table S13: Methylation variants within the same genes in pre-school children of the CHOP study (BMI) and adults in the study of Wahl. [3]

EWAS-BMI	CHOP						Wahl et al. 2017					
Gene	Rank # of 212 FDR sign.	CpG ID	CHR	Position (bp)	Change (SE) in BMI per % change in DNAm	uncorrected *P*-value	Rank # of 187 validated	CpG ID	CHR	Position (bp)	Change (SE) in BMI per % change * in DNAm	uncorrected *P*-value
ANKRD11	40	cg26666886	16	89558024	-0.32 (0.07)	3.37E-06	95	cg08726900	16	89550474	-0.10 (0.02)	2.00E-09
ANKRD11	59	cg27267258	16	89359175	0.35 (0.07)	5.06E-06	95	cg08726900	16	89550474	-0.10 (0.02)	2.00E-09
USP22	105	cg22078907	17	20920756	0.32 (0.07)	9.45E-06	121	cg25649826	17	20938740	0.17 (0.03)	8.90E-09
ZC3H3	125	cg13153009	8	144561626	-0.67 (0.15)	1.20E-05	57	cg26361535	8	144576604	0.11 (0.02)	1.40E-10

*) original estimate and SE were scaled to a 1% change by dividing orignal estimate by 100 to ease comparison with the CHOP study.

Supplementary Table S14: Methylation variants within the same genes in pre-school children of the CHOP study (FM) and adults in the study of Wahl. [3]

EWAS-FM	CHOP						Wahl et al. 2017					
Gene	Rank # of 230 FDR sign.	CpG ID	CHR	Position (bp)	Change (SE) in FM per % change in DNAm	uncorrected *P*-value	Rank # of 187 validated	CpG ID	CHR	Position (bp)	Change (SE) in BMI per % change * in DNAm	uncorrected *P*-value
ANKRD11	84	cg26666886	16	89558024	-0.25 (0.05)	7.43E-06	95	cg08726900	16	89550474	-0.10 (0.02)	2.00E-09
JARID2	68	cg14388804	6	15521099	0.25 (0.05)	5.54E-06	120	cg10975897	6	15504844	-0.13 (0.02)	8.20E-09
RPS6KA2	120	cg01577646	6	166911121	-0.13 (0.03)	1.26E-05	7	cg17501210	6	166970252	-0.18 (0.02)	8.20E-19
SLC41A1	202	cg26866142	1	205779678	0.35 (0.08)	2.28E-05	60	cg10717869	1	205780912	0.21 (0.03)	1.80E-10
ZC3H3	23	cg13153009	8	144561626	-0.59 (0.12)	1.28E-06	57	cg26361535	8	144576604	0.11 (0.02)	1.40E-10

*) original estimate and SE were scaled to a 1% change by dividing orignal estimate by 100 to ease comparison with the CHOP study.

Supplementary Table S15: Methylation variants within the same genes in pre-school children of the CHOP study (FMI) and adults in the study of Wahl. [3]

EWAS-FMI	CHOP						Wahl et al. 2017					
Gene	Rank # of 24 FDR sign.	CpG ID	CHR	Position (bp)	Change (SE) in FMI per % change in DNAm	uncorrected *P*-value	Rank # of 187 validated	CpG ID	CHR	Position (bp)	Change (SE) in BMI per % change * in DNAm	uncorrected *P*-value
SMC3	24	cg06322432	10	112289853	0.07 (0.01)	2.70E-06	159	cg26878209	10	112375475	0.15 (0.03)	4.30E-08

*) original estimate and SE were scaled to a 1% change by dividing orignal estimate by 100 to ease comparison with the CHOP study.

Supplementary Table S16: Methylation variants within the same genes in pre-school children of the CHOP study (FFM) and adults in the study of Wahl. [3]

EWAS-FFM	CHOP						Wahl et al. 2017					
Gene	Rank # of 120 FDR sign.	CpG ID	CHR	Position (bp)	Change (SE) in FFM per % change in DNAm	uncorrected *P*-value	Rank # of 187 validated	CpG ID	CHR	Position (bp)	Change (SE) in BMI per % change * in DNAm	uncorrected *P*-value
MAD1L1	85	cg05985070	7	2050170	-0.20 (0.04)	9.59E-06	85	cg05095590	7	2139259	0.10 (0.01)	9.6E-10
RPS6KA2	9	cg01577646	6	166911121	-0.21 (0.04)	6.85E-07	7	cg17501210	6	166970252	-0.18 (0.02)	8.2E-19
ZC3H3	58	cg25900150	8	144601851	-0.86 (0.19)	6.72E-06	57	cg26361535	8	144576604	0.11 (0.02)	1.4E-10

*) original estimate and SE were scaled to a 1% change by dividing orignal estimate by 100 to ease comparison with the CHOP study.

Supplementary Table S17: Best *P*-value rank of the associations of methylation variants within the *HIF3A* gene for all phenotypes in the pre-school children of the CHOP study.

Phenotype	EWAS best Rank	CpG ID	CHR	Position (bp)	Change (SE) in BMI per % change in DNAm	*P*-value	Bon-ferroni	FDR
BMI	30246	cg26749414	19	46807272	-0.0402 (0.0192)	3.73E-02	1	5.31E-01
ZBMI	36221	cg26749414	19	46807272	-0.0232 (0.0117)	4.89E-02	1	5.82E-01
FM	22602	cg26749414	19	46807272	-0.0354 (0.0154)	2.22E-02	1	4.23E-01
FMI	17577	cg26749414	19	46807272	-0.0247 (0.0105)	1.89E-02	1	4.63E-01
FFM	85950	cg02879662	19	46800467	-0.2309 (0.1475)	1.18E-01	1	5.94E-01
FFMI	38504	cg26749414	19	46807272	-0.0205 (0.0105)	5.11E-02	1	5.72E-01

Supplementary Table S18: *P*-value ranks of the associations of all methylation variants within the *HIF3A* gene and BMI in the pre-school children of the CHOP study.

Phenotype	EWAS Rank	CpG ID	CHR	Position (bp)	Change (SE) in BMI per % change in DNAm	*P*-value	Bon-ferroni	FDR
BMI	165898	cg07022477	19	46798869	-0.0312 (0.0319)	3.28E-01	1	8.54E-01
	152178	cg19045239	19	46799505	-0.0170 (0.0162)	2.96E-01	1	8.38E-01
	269330	cg15229275	19	46800054	0.0191 (0.0347)	5.84E-01	1	9.35E-01
	130965	cg19310908	19	46800064	-0.0717 (0.0617)	2.46E-01	1	8.09E-01
	185870	cg14117138	19	46800085	-0.0684 (0.0772)	3.76E-01	1	8.73E-01
	277155	cg10594090	19	46800198	0.0533 (0.1025)	6.04E-01	1	9.39E-01
	407942	cg14088357	19	46800338	-0.0066 (0.0865)	9.39E-01	1	9.93E-01
	325716	cg02879662	19	46800467	-0.0456 (0.1307)	7.28E-01	1	9.63E-01
	364305	cg09789590	19	46800479	-0.0105 (0.0476)	8.26E-01	1	9.78E-01
	243853	cg05286653	19	46800602	-0.0125 (0.0195)	5.20E-01	1	9.19E-01
	194475	cg27146050	19	46801557	-0.0164 (0.0193)	3.97E-01	1	8.81E-01
	293493	cg22891070	19	46801642	-0.0068 (0.0148)	6.45E-01	1	9.49E-01
	227199	cg16672562	19	46801672	-0.0096 (0.0135)	4.78E-01	1	9.08E-01
	187227	cg12068280	19	46804528	0.0188 (0.0213)	3.80E-01	1	8.75E-01
	416722	cg21617218	19	46805820	0.0060 (0.1269)	9.62E-01	1	9.96E-01
	125092	cg11253785	19	46806117	0.0842 (0.0704)	2.32E-01	1	8.01E-01
	300194	cg01552731	19	46806907	0.0170 (0.0390)	6.63E-01	1	9.52E-01
	263326	cg25196389	19	46806999	-0.0163 (0.0286)	5.69E-01	1	9.31E-01
	83817	cg23548163	19	46807119	-0.0152 (0.0103)	1.41E-01	1	7.25E-01
	**30246**	cg26749414	19	46807272	-0.0402 (0.0192)	3.73E-02	1	5.31E-01
	79344	cg20667364	19	46807466	0.0166 (0.0110)	1.32E-01	1	7.15E-01
	414754	cg07684068	19	46807660	0.0022 (0.0415)	9.57E-01	1	9.96E-01
	74716	cg25460031	19	46843271	0.0528 (0.0340)	1.22E-01	1	7.03E-01
	328141	cg14153927	19	46846465	0.0103 (0.0303)	7.34E-01	1	9.64E-01

Supplementary Table S19: mQTL – SNPs of the ARIES study for the FDR signifcant EWAS associations in pre-school children of the CHOP study (BMI).

EWAS-BMI	CHOP						mQTL (GCTA-database, Childhood)					ARIES[4]		
Rank # of 212 FDR sign.	CpG ID	CHR	Gene	Position (bp)	Change (SE) in BMI per % change in DNAm	*P*-value	SNP	SNP Chr	SNP Position (bp)	A1	A2	CpG -SNP association (beta)	*P*-value	mQTL Trans ?
34	cg24332767	3	*C3orf70*	184871524	0.15 (0.03)	2.83E-06	rs34580288	3	184874099	G	A	-0.4015	4.19E-10	N
53	cg01936370	3		156838174	0.09 (0.02)	4.41E-06	rs56268334	3	156848024	T	C	-0.4273	7.23E-19	N
60	cg08692210	3	*WDR51A*	52188851	0.18 (0.04)	5.11E-06	rs9816434	3	52197513	T	C	0.8139	4.42E-54	N
69	cg07664183	9	*HAUS6*	19103231	0.16 (0.03)	5.79E-06	rs7865126	9	19102828	C	T	-0.6415	9.09E-21	N
77	cg25371332	2		10688317	0.18 (0.04)	6.23E-06	rs12476576	2	10684530	C	G	0.3808	1.03E-20	N
111	cg24067118	10		102806933	0.08 (0.02)	1.11E-05	rs807035	10	102813193	A	G	0.5835	1.91E-46	N
138	cg19358373	12		133414571	0.11 (0.02)	1.35E-05	rs28495242	12	133447804	A	G	-0.5209	5.88E-28	N
148	cg19922435	4	*LOC285419*	124694178	0.06 (0.01)	1.44E-05	rs7660169	4	124655985	T	G	-0.7716	4.53E-49	N
158	cg02518775	11	*CNIH2*	66046770	0.13 (0.03)	1.57E-05	rs201239459	11	66016384	D	R	-1.495	4.56E-10	N
171	cg20380470	10		131987945	0.44 (0.10)	1.81E-05	rs79976723	10	131987377	A	G	0.6143	2.07E-23	N

Information on genetic variation using the ARIES GCTA-database can be found at http://mqtldb.org/. Search was restricted to time-point childhood with the default definition of 1000000+ bp for the distance of a trans or cis definition of an mQTL site. For details see Gaunt et al.[4]

Supplementary Table S20: mQTL – SNPs of the ARIES study for the FDR signifcant EWAS associations in pre-school children of the CHOP study (FM).

EWAS-FM	CHOP						mQTL (GCTA-database, Childhood)					ARIES[4]		
Rank # of 230 FDR sign.	CpG ID	CHR	Gene	Position (bp)	Change (SE) in BMI per % change in DNAm	*P*-value	SNP	SNP Chr	SNP Position (bp)	A1	A2	CpG -SNP association (beta)	*P*-value	mQTL Trans ?
2	cg08692210	3	*WDR51A*	52188851	0.17 (0.03)	5.89E-08	rs9816434	3	52197513	T	C	0.8139	4.42E-54	N
24	cg24332767	3	*C3orf70*	184871524	0.13 (0.03)	1.36E-06	rs34580288	3	184874099	G	A	-0.4015	4.19E-10	N
34	cg22430790	14	*FRMD6*	52117833	0.22 (0.05)	2.53E-06	rs34354107	14	52108652	D	R	0.3397	5.83E-24	N
66	cg22264170	6		9722973	-0.11 (0.02)	5.34E-06	rs138368004	6	9749733	D	R	-0.3858	6.54E-24	N
69	cg20380470	10		131987945	0.37 (0.08)	5.66E-06	rs79976723	10	131987377	A	G	0.6143	2.07E-23	N
77	cg07313882	17	*TMC6*	76121348	0.11 (0.02)	6.87E-06	rs34712518	17	76121031	T	C	0.4427	4.11E-10	N
82	cg19358373	12		133414571	0.09 (0.02)	7.21E-06	rs28495242	12	133447804	A	G	-0.5209	5.88E-28	N
91	cg07664183	9	*HAUS6*	19103231	0.13 (0.03)	8.75E-06	rs7865126	9	19102828	C	T	-0.6415	9.09E-21	N
205	cg24067118	10		102806933	0.06 (0.01)	2.29E-05	rs807035	10	102813193	A	G	0.5835	1.91E-46	N
206	cg25371332	2		10688317	0.14 (0.03)	2.31E-05	rs12476576	2	10684530	C	G	0.3808	1.03E-20	N
225	cg21309845	17	*TMEM95*	7258498	0.14 (0.03)	2.55E-05	rs72840988	17	7258481	T	C	0.2619	5.72E-12	N
228	cg15923936	6		91318412	0.09 (0.02)	2.63E-05	rs398026	6	91318768	G	A	-0.3854	3.46E-22	N

Information on genetic variation using the ARIES GCTA-database can be found at http://mqtldb.org/. Search was restricted to time-point childhood with the default definition of 1000000+ bp for the distance of a trans or cis definition of an mQTL site. For details see Gaunt et al.[4]

Supplementary Table S21: mQTL – SNPs of the ARIES study for the FDR signifcant EWAS associations in pre-school children of the CHOP study (FMI).

EWAS-FMI	CHOP						mQTL (GCTA-database, childhood)					ARIES[4]		
Rank # of 24 FDR sign.	CpG ID	CHR	Gene	Position (bp)	Change (SE) in BMI per % change in DNAm	*P*-value	SNP	SNP Chr	SNP Position (bp)	A1	A2	CpG -SNP association (beta)	*P*-value	mQTL Trans ?
9	cg08692210	3	*WDR51A*	52188851	0.11 (0.02)	2.95E-07	rs9816434	3	52197513	T	C	0.8139	4.42E-54	N
21	cg22264170	6	*MRDS1;OFCC1*	9722973	-0.07 (0.02)	2.39E-06	rs138368004	6	9749733	D	R	-0.3858	6.54E-24	N

Information on genetic variation using the ARIES GCTA-database can be found at http://mqtldb.org/. Search was restricted to time-point childhood with the default definition of 1000000+ bp for the distance of a trans or cis definition of an mQTL site. For details see Gaunt et al.[4]

Supplementary Table S22: mQTL – SNPs of the ARIES study for the FDR signifcant EWAS associations in pre-school children of the CHOP study (FFM).

EWAS-FFM	CHOP						mQTL (GCTA-database, childhood)					ARIES[4]		
**Rank # of 120 FDR sign.**	**CpG ID**	**CHR**	**Gene**	**Position (bp)**	**Change (SE) in BMI per % change in DNAm**	***P*-value**	**SNP**	**SNP Chr**	**SNP Position (bp)**	**A1**	**A2**	**CpG -SNP association (beta)**	***P*-value**	**mQTL Trans ?**
3	cg24332767	3	*C3orf70*	184871524	0.20 (0.04)	3.70E-08	rs34580288	3	184874099	G	A	-0.4015	4.19E-10	N
32	cg25901204	15	*ISL2*	76628858	0.22 (0.05)	3.45E-06	rs3866543	15	76629609	T	G	0.2828	1.09E-15	N
34	cg25371332	2		10688317	0.21 (0.04)	3.59E-06	rs12476576	2	10684530	C	G	0.3808	1.03E-20	N
36	cg02532700	22	*NCF4*	37257404	0.11 (0.02)	3.68E-06	rs4821544	22	37258503	C	T	0.5759	1.27E-50	N
49	cg20380470	10		131987945	0.52 (0.11)	5.32E-06	rs79976723	10	131987377	A	G	0.6143	2.07E-23	N
65	cg19903596	5		180527369	0.11 (0.02)	7.68E-06	rs4355482	5	180527367	A	G	-0.6221	1.92E-62	N
82	cg02736908	17		29887376	0.10 (0.02)	9.40E-06	rs1548470	17	29862862	A	G	0.418	7.78E-24	N
99	cg06452513	17	*UNC119*	26879897	0.16 (0.04)	1.12E-05	rs652677	17	26904517	A	C	0.4788	5.32E-29	N
100	cg00170487	4	*FHDC1*	153871345	0.11 (0.03)	1.14E-05	rs12510260	4	153871340	T	C	-0.4221	6.67E-30	N

Information on genetic variation using the ARIES GCTA-database can be found at http://mqtldb.org/. Search was restricted to time-point childhood with the default definition of 1000000+ bp for the distance of a trans or cis definition of an mQTL site. For details see Gaunt et al.[4]

Supplementary Table S23: Percent change in DNA-methylation by BMI unit (kg/m^2) for the top 50 probes from the reference free EWAS model (K=9).

Rank	CpG ID	CHR	Gene	UCSC REFGENE GROUP	RELATION TO UCSC CPG ISLAND	Position (bp)	% DNAm Change (SE) per BMI unit	*P*-value	Bon-ferroni	FDR
1	cg13850887	2	*SNED1*	Body	S_Shore	241993303	-0.43 (0.07)	3.17E-10	1.37E-04	1.37E-04
2	cg04010122	2	*SOS1*	Body	N_Shore	39346883	0.38 (0.06)	3.13E-09	1.35E-03	6.76E-04
3	cg08692210	3	*WDR51A*	TSS200;TSS200;TSS200	Island	52188851	0.36 (0.06)	1.10E-08	4.75E-03	1.58E-03
4	cg26627956	2	*CFLAR*	Body;Body;Body		202004766	-0.34 (0.06)	3.13E-08	1.35E-02	3.38E-03
5	cg06594770	22	*TRIOBP*	TSS200		38092974	0.16 (0.03)	1.32E-07	5.69E-02	1.14E-02
6	cg02038492	16	*CASKIN1*	Body	N_Shore	2235025	0.36 (0.07)	2.69E-07	1.16E-01	1.82E-02
7	cg04219544	17	*KRT24*	TSS200		38860042	-0.49 (0.09)	2.95E-07	1.27E-01	1.82E-02
8	cg08541599	19	*BSG*	Body;Body;Body	Island	579501	-0.43 (0.08)	3.64E-07	1.57E-01	1.95E-02
9	cg17203352	17	*WFIKKN2*	TSS200		48912487	-0.44 (0.09)	4.06E-07	1.75E-01	1.95E-02
10	cg02518775	11	*CNIH2*	Body	S_Shore	66046770	0.41 (0.08)	1.09E-06	4.70E-01	4.51E-02
11	cg15026574	3	*ST6GAL1*	5'UTR;5'UTR		186683429	-0.38 (0.08)	1.18E-06	5.10E-01	4.51E-02
12	cg26401512	1	*ZNF643*	Body		40922773	-0.42 (0.09)	1.36E-06	5.86E-01	4.51E-02
13	cg22264170	6				9722973	-0.44 (0.09)	1.36E-06	5.87E-01	4.51E-02
14	cg02682525	11	*ANKK1*	TSS1500	N_Shore	113258277	0.42 (0.09)	1.62E-06	6.99E-01	4.99E-02
15	cg12379720	22	*TRIOBP*	TSS200		38092989	0.26 (0.05)	1.97E-06	8.52E-01	5.68E-02
16	cg19358373	12			S_Shore	133414571	0.54 (0.11)	2.14E-06	9.24E-01	5.78E-02
17	cg14834653	10	*FGFR2*	TSS200;5'UTR;5'UTR;TSS1500;5'UTR; TSS1500;5'UTR;5'UTR;5'UTR	N_Shelf	123353791	-0.29 (0.06)	2.50E-06	1	6.34E-02
18	cg26867987	6	*COL11A2*	3'UTR;3'UTR;3'UTR	S_Shore	33131450	-0.44 (0.09)	2.87E-06	1	6.67E-02
19	cg16810626	8	*GATA4*	Body		11594003	0.89 (0.19)	2.99E-06	1	6.67E-02
20	cg07764575	19	*CATSPERG*	Body	N_Shore	38850992	-0.56 (0.12)	3.15E-06	1	6.67E-02
21	cg19922435	4	*LOC285419*	Body;TSS1500		124694178	0.84 (0.18)	3.25E-06	1	6.67E-02
22	cg09245901	6				132075947	0.66 (0.14)	3.61E-06	1	7.07E-02
23	cg26882525	5	*SLC36A3*	Body;Body		150677867	-3.10 (0.67)	4.82E-06	1	8.71E-02
24	cg05911774	X	*SRPX2*	1stExon;5'UTR		99899345	0.32 (0.07)	4.85E-06	1	8.71E-02
25	cg05120716	16	*PRKCB*	Body;Body		23881993	-0.45 (0.10)	5.78E-06	1	9.98E-02
26	cg12610207	12	*TBX5*	Body;Body;Body;Body	Island	114833950	0.08 (0.02)	6.43E-06	1	1.07E-01
27	cg01184539	6	*FAM26D;BET3L*	TSS1500;Body		116848950	0.31 (0.07)	7.08E-06	1	1.11E-01

28	cg01166564	2	*C2orf18*	Body	Island	26987492	0.12 (0.03)	7.21E-06	1	1.11E-01
29	cg14124917	2	*GRHL1*	TSS1500	Island	10090974	0.20 (0.04)	8.99E-06	1	1.31E-01
30	cg01941047	5	*SFRS12*	5'UTR;Body;1stExon	Island	65440763	-0.06 (0.01)	9.10E-06	1	1.31E-01
31	cg20122727	4	*BTC*	TSS200	S_Shore	75719957	0.46 (0.10)	1.08E-05	1	1.46E-01
32	cg23611697	2				102666444	-0.36 (0.08)	1.09E-05	1	1.46E-01
33	cg20655548	11	*TRIM29*	1stExon	N_Shore	120008025	-0.24 (0.05)	1.12E-05	1	1.46E-01
34	cg24067118	10			N_Shore	102806933	0.60 (0.13)	1.24E-05	1	1.53E-01
35	cg01178624	11	*KCNK7*	3'UTR;3'UTR;3'UTR;3'UTR	Island	65360327	1.17 (0.26)	1.27E-05	1	1.53E-01
36	cg22798261	19	*THEG;THEG*	TSS1500;TSS1500	S_Shore	376653	-1.27 (0.29)	1.27E-05	1	1.53E-01
37	cg17810765	2	*ANO7*	TSS200;TSS200		242127836	0.39 (0.09)	1.37E-05	1	1.60E-01
38	cg05715492	7	*ARPC1B*	Body	S_Shore	98991138	0.91 (0.21)	1.45E-05	1	1.64E-01
39	cg01127586	X	*LOC92249*	TSS200		62780937	-0.50 (0.11)	1.48E-05	1	1.64E-01
40	cg01127608	9	*LMX1B*	Body	Island	129377854	3.51 (0.80)	1.54E-05	1	1.67E-01
41	cg14783904	17	*GLP2R*	1stExon		9729422	0.56 (0.13)	1.85E-05	1	1.91E-01
42	cg00250743	10	*FGFR2*	Body;Body;Body;Body;Body;Body;Body;Body;Body		123264130	-0.40 (0.09)	1.86E-05	1	1.91E-01
43	cg14398659	11	*INTS4*	TSS200	Island	77705832	-0.07 (0.02)	1.96E-05	1	1.96E-01
44	cg20776385	15			N_Shore	40613333	0.25 (0.06)	2.21E-05	1	2.17E-01
45	cg10119082	7	*ARPC1B*	Body	Island	98990626	0.49 (0.11)	2.43E-05	1	2.33E-01
46	cg06376715	1	*TP73*	Body;Body;Body;Body	Island	3634918	0.50 (0.12)	2.71E-05	1	2.47E-01
47	cg10277500	15	*LYSMD2;TMOD2*	1stExon;TSS1500;TSS1500;5'UTR	Island	52043553	0.08 (0.02)	2.74E-05	1	2.47E-01
48	cg06644488	22	*TRIOBP*	TSS200		38092896	0.18 (0.04)	2.76E-05	1	2.47E-01
49	cg11072119	1	*RAP1GAP*	Body;Body;Body	S_Shelf	21934312	0.31 (0.07)	2.80E-05	1	2.47E-01
50	cg14289863	9	*SMC5*	TSS1500	N_Shore	72872391	-0.30 (0.07)	3.19E-05	1	2.75E-01

Genes in brackets indicate manual annotation for closest genes using the UCSC genome browser on human GRCh37/hg19 assembly. The record highlighted in red indicates the last methylation site significantly associated with the outcome acccording to Bonferroni correction and the blue highlighted record the last signficant according to false-discovery rate.[5] The estimates and *P*-values are based on the reference free EWAS model of Houseman [6,7]with DNAm as outcome and phenotype, all covariates inclusive 30 control probe PCs and K=9 latent dimensions instead of 6 cell mix adjustment as independent variables. Inflation due to genomic control[8] is 0.761 and is 0.197 according to van Iterson's "bacon-method". [9]

Supplementary Table S24: Percent change in DNA-methylation by ZBMI unit (z-score) for the top 50 probes from the reference free EWAS model (K=9).

Rank	CpG ID	CHR	Gene	UCSC REFGENE GROUP	RELATION TO UCSC CPG ISLAND	Position (bp)	% DNAm Change (SE) per ZBMI unit	*P*-value	Bon-ferroni	FDR
1	cg04010122	2	*SOS1*	Body	N_Shore	39346883	0.57 (0.09)	6.58E-10	2.84E-04	2.84E-04
2	cg13850887	2	*SNED1*	Body	S_Shore	241993303	-0.67 (0.11)	3.03E-09	1.31E-03	6.20E-04
3	cg08692210	3	*WDR51A*	TSS200;TSS200;TSS200	Island	52188851	0.52 (0.09)	4.31E-09	1.86E-03	6.20E-04
4	cg26867987	6	*COL11A2*	3'UTR;3'UTR;3'UTR	S_Shore	33131450	-0.65 (0.11)	1.95E-08	8.40E-03	2.10E-03
5	cg09245901	6				132075947	1.08 (0.19)	4.11E-08	1.77E-02	3.50E-03
6	cg06594770	22	*TRIOBP*	TSS200		38092974	0.23 (0.04)	5.54E-08	2.39E-02	3.50E-03
7	cg26627956	2	*CFLAR*	Body;Body;Body		202004766	-0.52 (0.09)	5.69E-08	2.45E-02	3.50E-03
8	cg18287222	6	*MAP3K5*	Body		137059970	0.51 (0.10)	1.76E-07	7.59E-02	9.49E-03
9	cg26401512	1	*ZNF643*	Body		40922773	-0.62 (0.12)	2.68E-07	1.15E-01	1.28E-02
10	cg20301680	1	*SSBP3*	Body;Body;Body		54786494	0.45 (0.09)	4.36E-07	1.88E-01	1.88E-02
11	cg04219544	17	*KRT24*	TSS200		38860042	-0.80 (0.16)	8.50E-07	3.66E-01	3.03E-02
12	cg07764575	19	*CATSPERG*	Body	N_Shore	38850992	-0.84 (0.17)	9.40E-07	4.06E-01	3.03E-02
13	cg01166564	2	*C2orf18*	Body	Island	26987492	0.19 (0.04)	9.75E-07	4.21E-01	3.03E-02
14	cg14289863	9	*SMC5*	TSS1500	N_Shore	72872391	-0.47 (0.09)	9.84E-07	4.25E-01	3.03E-02
15	cg17203352	17	*WFIKKN2*	TSS200		48912487	-0.66 (0.13)	1.09E-06	4.69E-01	3.13E-02
16	cg15026574	3	*ST6GAL1*	5'UTR;5'UTR		186683429	-0.57 (0.12)	1.35E-06	5.84E-01	3.65E-02
17	cg19358373	12			S_Shore	133414571	0.86 (0.18)	1.70E-06	7.34E-01	4.32E-02
18	cg12379720	22	*TRIOBP*	TSS200		38092989	0.40 (0.08)	2.69E-06	1	6.44E-02
19	cg22798261	19	*THEG*	TSS1500;TSS1500	S_Shore	376653	-1.86 (0.40)	3.90E-06	1	8.86E-02
20	cg00955911	14	*FOXA1*	Body	Island	38061988	0.38 (0.08)	4.75E-06	1	1.03E-01
21	cg24102266	1	*KIAA1522*	Body	Island	33219584	-0.09 (0.02)	5.84E-06	1	1.19E-01
22	cg10962535	19	*ITGB1BP3*	5'UTR	Island	3933474	0.32 (0.07)	6.05E-06	1	1.19E-01
23	cg20058148	4	*MAP9*	TSS1500	S_Shore	156298427	-0.96 (0.21)	7.47E-06	1	1.40E-01
24	cg22264170	6				9722973	-0.64 (0.14)	8.00E-06	1	1.44E-01
25	cg14834653	10	*FGFR2*	TSS200;5'UTR;5'UTR;TSS1500;5'UTR; TSS1500;5'UTR;5'UTR;5'UTR	N_Shelf	123353791	-0.46 (0.10)	9.83E-06	1	1.59E-01
26	cg24067118	10			N_Shore	102806933	1.02 (0.23)	9.93E-06	1	1.59E-01

27	cg20860188	2	*KLHL29*	5'UTR	S_Shelf	23613280	-1.14 (0.25)	9.97E-06	1	1.59E-01
28	cg14621090	13				113571679	-0.55 (0.12)	1.06E-05	1	1.64E-01
29	cg14398659	11	*INTS4*	TSS200	Island	77705832	-0.11 (0.02)	1.13E-05	1	1.68E-01
30	cg19922435	4	*LOC285419*	Body;TSS1500		124694178	1.37 (0.31)	1.23E-05	1	1.76E-01
31	cg26882525	5	*SLC36A3*	Body;Body		150677867	-5.15 (1.17)	1.34E-05	1	1.86E-01
32	cg17378617	11	*NXF1*	Body;Body	N_Shore	62572592	0.30 (0.07)	1.60E-05	1	2.15E-01
33	cg05120716	16	*PRKCB*	Body;Body		23881993	-0.67 (0.15)	1.69E-05	1	2.15E-01
34	cg10788371	11	*LRRC32*	5'UTR;1stExon;5'UTR	N_Shore	76381040	0.63 (0.14)	1.73E-05	1	2.15E-01
35	cg02682525	11	*ANKK1*	TSS1500	N_Shore	113258277	0.66 (0.15)	1.74E-05	1	2.15E-01
36	cg05715492	7	*ARPC1B*	Body	S_Shore	98991138	1.40 (0.32)	1.83E-05	1	2.19E-01
37	cg17082365	X	*ARHGAP6*	Body;Body	Island	11157323	-1.94 (0.45)	1.88E-05	1	2.19E-01
38	cg03214277	19	*SLC25A23*	TSS1500	Island	6460154	-0.13 (0.03)	2.01E-05	1	2.28E-01
39	cg23300732	4	*STK32B*	TSS200	Island	5053496	-0.14 (0.03)	2.10E-05	1	2.31E-01
40	cg24314057	1	*RGS2*	TSS200	Island	192778043	0.08 (0.02)	2.16E-05	1	2.31E-01
41	cg12610207	12	*TBX5*	Body;Body;Body;Body	Island	114833950	0.13 (0.03)	2.23E-05	1	2.31E-01
42	cg01976623	14	*PRIMA1*	TSS1500	S_Shore	94255901	0.80 (0.19)	2.25E-05	1	2.31E-01
43	cg00250743	10	*FGFR2*	Body;Body;Body;Body;Body;<br>Body;Body;Body;Body		123264130	-0.60 (0.14)	2.31E-05	1	2.32E-01
44	cg23470262	12	*GOLGA3*	Body		133383213	0.42 (0.10)	2.48E-05	1	2.43E-01
45	cg11701951	12	*CSRNP2*	5'UTR;1stExon	Island	51477122	-0.10 (0.02)	2.86E-05	1	2.73E-01
46	cg24093300	7	*PTPRN2*	Body;Body;Body		157543805	-0.47 (0.11)	2.91E-05	1	2.73E-01
47	cg16392111	1				55218005	0.34 (0.08)	3.03E-05	1	2.78E-01
48	cg02518775	11	*CNIH2*	Body	S_Shore	66046770	0.65 (0.15)	3.09E-05	1	2.78E-01
49	cg21594646	15	*PIGB*	5'UTR;1stExon	Island	55611305	-0.17 (0.04)	3.38E-05	1	2.94E-01
50	cg16072030	1	*PPP1R15B*	TSS1500	Island	204381148	-0.10 (0.02)	3.41E-05	1	2.94E-01

Genes in brackets indicate manual annotation for closest genes using the UCSC genome browser on human GRCh37/hg19 assembly. The record highlighted in red indicates the last methylation site significantly associated with the outcome acccording to Bonferroni correction and the blue highlighted record the last signficant according to false-discovery rate.[5] The estimates and *P*-values are based on the reference free EWAS model of Houseman [6,7] with DNAm as outcome and phenotype, all covariates inclusive 30 control probe PCs and K=9 latent dimensions instead of 6 cell mix adjustment as independent variables. Inflation due to genomic control[8] is 0.756 and is 0.197 according to van Iterson's "bacon-method". [9]

Supplementary Table S25: Percent change in DNA-methylation by FM unit (kg) for the top 50 probes from the reference free EWAS model (K=9).

Rank	CpG ID	CHR	Gene	UCSC REFGENE GROUP	RELATION TO UCSC CPG ISLAND	Position (bp)	% DNAm Change (SE) per FM unit	*P*-value	Bon-ferroni	FDR
1	cg13850887	2	*SNED1*	Body	S_Shore	241993303	-0.61 (0.09)	1.25E-11	5.37E-06	5.37E-06
2	cg08692210	3	*WDR51A*	TSS200;TSS200;TSS200	Island	52188851	0.51 (0.08)	1.26E-10	5.45E-05	2.73E-05
3	cg04010122	2	*SOS1*	Body	N_Shore	39346883	0.55 (0.09)	3.64E-09	1.57E-03	4.87E-04
4	cg26627956	2	*CFLAR*	Body;Body;Body		202004766	-0.49 (0.08)	4.51E-09	1.95E-03	4.87E-04
5	cg05120716	16	*PRKCB*	Body;Body		23881993	-0.63 (0.11)	7.20E-09	3.10E-03	6.21E-04
6	cg26867987	6	*COL11A2*	3'UTR;3'UTR;3'UTR	S_Shore	33131450	-0.54 (0.09)	3.25E-08	1.40E-02	2.34E-03
7	cg17203352	17	*WFIKKN2*	TSS200		48912487	-0.59 (0.11)	5.44E-08	2.35E-02	2.81E-03
8	cg22264170	6				9722973	-0.67 (0.12)	5.53E-08	2.39E-02	2.81E-03
9	cg22798261	19	*THEG*	TSS1500;TSS1500	S_Shore	376653	-1.47 (0.27)	6.38E-08	2.75E-02	2.81E-03
10	cg08541599	19	*BSG*	Body;Body;Body	Island	579501	-0.66 (0.12)	6.78E-08	2.92E-02	2.81E-03
11	cg01166564	2	*C2orf18*	Body	Island	26987492	0.17 (0.03)	7.16E-08	3.09E-02	2.81E-03
12	cg15026574	3	*ST6GAL1*	5'UTR;5'UTR		186683429	-0.49 (0.09)	8.31E-08	3.58E-02	2.99E-03
13	cg06594770	22	*TRIOBP*	TSS200		38092974	0.21 (0.04)	1.02E-07	4.39E-02	3.38E-03
14	cg02317742	2	*ATOH8*	1stExon;5'UTR	Island	85981021	0.12 (0.02)	1.58E-07	6.83E-02	4.88E-03
15	cg12379720	22	*TRIOBP*	TSS200		38092989	0.34 (0.06)	2.22E-07	9.59E-02	6.40E-03
16	cg12403162	10	*ABLIM1*	Body;Body;Body	S_Shelf	116394557	-0.58 (0.11)	3.01E-07	1.30E-01	8.12E-03
17	cg23611697	2				102666444	-0.58 (0.11)	5.53E-07	2.39E-01	1.40E-02
18	cg07894717	1	*APITD1;CORT*	Body;TSS1500		10508598	-0.52 (0.10)	5.98E-07	2.58E-01	1.43E-02
19	cg26401512	1	*ZNF643*	Body		40922773	-0.49 (0.10)	8.63E-07	3.72E-01	1.96E-02
20	cg01184539	6	*FAM26D;BET3L*	TSS1500;Body		116848950	0.48 (0.10)	1.26E-06	5.42E-01	2.71E-02
21	cg14621090	13				113571679	-0.53 (0.11)	2.41E-06	1	4.74E-02
22	cg08936794	1	*ELOVL1*	TSS200	Island	43833873	0.17 (0.04)	2.42E-06	1	4.74E-02
23	cg19358373	12			S_Shore	133414571	0.70 (0.15)	3.03E-06	1	5.68E-02
24	cg02853524	3				145768808	-0.66 (0.14)	3.60E-06	1	6.48E-02
25	cg06644488	22	*TRIOBP*	TSS200		38092896	0.23 (0.05)	3.75E-06	1	6.48E-02
26	cg24999308	15	*NPTN*	1stExon;1stExon;1stExon;1stExon	Island	73925545	0.12 (0.02)	4.83E-06	1	8.01E-02
27	cg09357438	4	*SPINK2*	TSS1500	S_Shore	57688282	-0.31 (0.07)	5.89E-06	1	9.41E-02

28	cg22062432	5			S_Shore	180634502	0.31 (0.07)	6.36E-06	1	9.80E-02
29	cg07652350	11	*IRF7*	TSS1500;TSS200;TSS200	Island	616009	-0.10 (0.02)	8.48E-06	1	1.25E-01
30	cg14124917	2	*GRHL1*	TSS1500	Island	10090974	0.25 (0.05)	8.68E-06	1	1.25E-01
31	cg24102266	1	*KIAA1522*	Body	Island	33219584	-0.06 (0.01)	9.44E-06	1	1.25E-01
32	cg05063339	4			Island	54957980	-0.09 (0.02)	9.53E-06	1	1.25E-01
33	cg27038634	8			N_Shore	49468341	0.65 (0.15)	9.80E-06	1	1.25E-01
34	cg14397361	9	*COBRA1*	1stExon	Island	140149997	0.11 (0.02)	9.85E-06	1	1.25E-01
35	cg01127586	X	*LOC92249*	TSS200		62780937	-0.59 (0.13)	1.08E-05	1	1.31E-01
36	cg10483909	1	*CEP170;SDCCAG8*	5'UTR;1stExon;1stExon; TSS1500;1stExon;5'UTR;5'UTR	Island	243418637	0.17 (0.04)	1.10E-05	1	1.31E-01
37	cg06917785	19	*KANK3*	TSS200	Island	8408327	0.19 (0.04)	1.14E-05	1	1.31E-01
38	cg14268632	22	*TRIOBP*	TSS200		38092830	0.23 (0.05)	1.17E-05	1	1.31E-01
39	cg20856330	6	*RPP21*	Body	S_Shore	30313546	-0.63 (0.14)	1.18E-05	1	1.31E-01
40	cg09590733	4	*DSPP*	5'UTR		88530371	0.37 (0.08)	1.50E-05	1	1.55E-01
41	cg19360425	19	*PTPRS*	Body;Body;Body;Body	S_Shore	5213208	-0.44 (0.10)	1.52E-05	1	1.55E-01
42	cg04310104	6	*TFAP2B*	TSS1500	N_Shore	50785757	-0.35 (0.08)	1.54E-05	1	1.55E-01
43	cg09121478	1	*SLC2A7*	TSS200		9086460	-0.51 (0.12)	1.57E-05	1	1.55E-01
44	cg26872151	10	*PIP4K2A*	Body		22908265	-0.74 (0.17)	1.62E-05	1	1.55E-01
45	cg07327568	4	*CLOCK*	5'UTR		56393876	0.39 (0.09)	1.67E-05	1	1.55E-01
46	cg13870041	1				227015885	0.24 (0.05)	1.68E-05	1	1.55E-01
47	cg20093577	11	*SF3B2*	TSS200	Island	65819742	0.08 (0.02)	1.75E-05	1	1.55E-01
48	cg07764575	19	*CATSPERG*	Body	N_Shore	38850992	-0.70 (0.16)	1.82E-05	1	1.55E-01
49	cg11350053	6	*IER3;FLOT1*	3'UTR;TSS1500	Island	30711028	0.32 (0.07)	1.85E-05	1	1.55E-01
50	cg24093300	7	*PTPRN2*	Body;Body;Body		157543805	-0.42 (0.10)	1.85E-05	1	1.55E-01

Genes in brackets indicate manual annotation for closest genes using the UCSC genome browser on human GRCh37/hg19 assembly. The record highlighted in red indicates the last methylation site significantly associated with the outcome acccording to Bonferroni correction and the blue highlighted record the last signficant according to false-discovery rate.[5] The estimates and *P*-values are based on the reference free EWAS model of Houseman [6,7]with DNAm as outcome and phenotype, all covariates inclusive 30 control probe PCs and K=9 latent dimensions instead of 6 cell mix adjustment as independent variables. Inflation due to genomic control is 0.775 and is 0.203 according to van Iterson's "bacon-method". [9]

Supplementary Table S26: Percent change in DNA-methylation by FMI unit (kg/m²) for the top 50 probes from the reference free EWAS model (K=9).

Rank	CpG ID	CHR	Gene	UCSC REFGENE GROUP	RELATION TO UCSC CPG ISLAND	Position (bp)	% DNAm Change (SE) per FMI unit	*P*-value	Bon-ferroni	FDR
1	cg08692210	3	*WDR51A*	TSS200;TSS200;TSS200	Island	52188851	0.72 (0.10)	5.66E-13	2.44E-07	2.44E-07
2	cg13850887	2	*SNED1*	Body	S_Shore	241993303	-0.94 (0.13)	4.43E-12	1.91E-06	9.55E-07
3	cg26627956	2	*CFLAR*	Body;Body;Body		202004766	-0.74 (0.11)	1.31E-11	5.67E-06	1.89E-06
4	cg06594770	22	*TRIOBP*	TSS200		38092974	0.31 (0.05)	3.98E-11	1.72E-05	4.29E-06
5	cg22264170	6				9722973	-1.02 (0.16)	4.26E-10	1.84E-04	3.68E-05
6	cg17203352	17	*WFIKKN2*	TSS200		48912487	-0.90 (0.15)	1.70E-09	7.34E-04	1.22E-04
7	cg26867987	6	*COL11A2*	3'UTR;3'UTR;3'UTR	S_Shore	33131450	-0.82 (0.13)	2.57E-09	1.11E-03	1.58E-04
8	cg01166564	2	*C2orf18*	Body	Island	26987492	0.25 (0.04)	7.37E-09	3.18E-03	3.97E-04
9	cg07894717	1	*APITD1;CORT*	Body;TSS1500		10508598	-0.82 (0.14)	1.28E-08	5.50E-03	5.98E-04
10	cg17378617	11	*NXF1;NXF1*	Body;Body	N_Shore	62572592	0.42 (0.07)	1.39E-08	5.98E-03	5.98E-04
11	cg07764575	19	*CATSPERG*	Body	N_Shore	38850992	-1.14 (0.20)	1.64E-08	7.06E-03	6.42E-04
12	cg09121478	1	*SLC2A7*	TSS200		9086460	-0.78 (0.14)	2.36E-08	1.02E-02	8.49E-04
13	cg14621090	13				113571679	-0.79 (0.14)	3.66E-08	1.58E-02	1.21E-03
14	cg26401512	1	*ZNF643*	Body		40922773	-0.78 (0.14)	5.39E-08	2.33E-02	1.66E-03
15	cg12379720	22	*TRIOBP*	TSS200		38092989	0.51 (0.09)	1.07E-07	4.63E-02	3.09E-03
16	cg15026574	3	*ST6GAL1*	5'UTR;5'UTR		186683429	-0.74 (0.14)	1.44E-07	6.19E-02	3.87E-03
17	cg22798261	19	*THEG*	TSS1500;TSS1500	S_Shore	376653	-2.31 (0.43)	1.79E-07	7.74E-02	4.55E-03
18	cg24093300	7	*PTPRN2*	Body;Body;Body		157543805	-0.64 (0.12)	2.90E-07	1.25E-01	6.94E-03
19	cg06644488	22	*TRIOBP*	TSS200		38092896	0.35 (0.07)	3.79E-07	1.64E-01	8.61E-03
20	cg06825849	16	*LOC342346*	Body		4635594	0.31 (0.06)	4.28E-07	1.84E-01	9.22E-03
21	cg23090396	1	*MEGF6*	Body		3407936	-0.16 (0.03)	1.23E-06	5.30E-01	2.52E-02
22	cg22561727	19	*DPP9*	Body	N_Shelf	4719848	-0.92 (0.19)	1.75E-06	7.57E-01	3.44E-02
23	cg01184539	6	*FAM26D;BET3L*	TSS1500;Body		116848950	0.66 (0.14)	1.90E-06	8.19E-01	3.54E-02
24	cg14398659	11	*INTS4*	TSS200	Island	77705832	-0.12 (0.02)	2.04E-06	8.79E-01	3.54E-02
25	cg00955911	14	*FOXA1*	Body	Island	38061988	0.46 (0.10)	2.05E-06	8.84E-01	3.54E-02
26	cg23052191	8	*AZIN1*	TSS200;TSS200	Island	103876402	-0.12 (0.02)	2.55E-06	1	4.13E-02
27	cg23611697	2				102666444	-0.82 (0.17)	2.72E-06	1	4.13E-02

28	cg14334161	10	*NODAL*	TSS200	S_Shore	72201622	0.98 (0.20)	2.73E-06	1	4.13E-02
29	cg20776385	15			N_Shore	40613333	0.48 (0.10)	2.78E-06	1	4.13E-02
30	cg19358373	12			S_Shore	133414571	0.97 (0.20)	2.96E-06	1	4.15E-02
31	cg01127608	9	*LMX1B*	Body	Island	129377854	7.56 (1.59)	2.99E-06	1	4.15E-02
32	cg12403162	10	*ABLIM1*	Body;Body;Body	S_Shelf	116394557	-0.89 (0.19)	3.49E-06	1	4.71E-02
33	cg05120716	16	*PRKCB*	Body;Body		23881993	-0.95 (0.20)	4.48E-06	1	5.86E-02
34	cg23681745	2	*CFLAR*	Body;Body;Body		202004875	-0.86 (0.19)	4.72E-06	1	5.99E-02
35	cg09245901	6				132075947	1.00 (0.22)	5.95E-06	1	7.31E-02
36	cg08541599	19	*BSG*	Body;Body;Body	Island	579501	-0.96 (0.21)	6.10E-06	1	7.31E-02
37	cg10877187	10	*LIPN*	Body		90529889	-0.76 (0.17)	6.45E-06	1	7.51E-02
38	cg04219544	17	*KRT24*	TSS200		38860042	-0.81 (0.18)	7.80E-06	1	8.85E-02
39	cg24102266	1	*KIAA1522*	Body	Island	33219584	-0.09 (0.02)	8.43E-06	1	9.02E-02
40	cg14289863	9	*SMC5*	TSS1500	N_Shore	72872391	-0.51 (0.11)	8.60E-06	1	9.02E-02
41	cg16726039	12			Island	133413162	1.37 (0.30)	8.75E-06	1	9.02E-02
42	cg22062432	5			S_Shore	180634502	0.48 (0.11)	8.80E-06	1	9.02E-02
43	cg11084035	11	*GANAB*	1stExon;1stExon	Island	62414045	-0.08 (0.02)	8.99E-06	1	9.02E-02
44	cg16115720	1	*CD34*	1stExon;1stExon	Island	208084415	0.10 (0.02)	9.52E-06	1	9.23E-02
45	cg02682525	11	*ANKK1*	TSS1500	N_Shore	113258277	0.78 (0.17)	9.70E-06	1	9.23E-02
46	cg05691152	22	*TRIOBP*	TSS200		38092978	0.43 (0.10)	9.84E-06	1	9.23E-02
47	cg02853524	3				145768808	-0.97 (0.22)	1.06E-05	1	9.77E-02
48	cg12610207	12	*TBX5*	Body;Body;Body;Body	Island	114833950	0.14 (0.03)	1.12E-05	1	9.92E-02
49	cg15809858	5	*PPARGC1B*	Body	Island	149111017	0.79 (0.18)	1.13E-05	1	9.92E-02
50	cg26872151	10	*PIP4K2A*	Body		22908265	-1.21 (0.27)	1.16E-05	1	1.00E-01

Genes in brackets indicate manual annotation for closest genes using the UCSC genome browser on human GRCh37/hg19 assembly. The record highlighted in red indicates the last methylation site significantly associated with the outcome acccording to Bonferroni correction and the blue highlighted record the last signficant according to false-discovery rate.[5] The estimates and *P*-values are based on the reference free EWAS model of Houseman [6,7]with DNAm as outcome and phenotype, all covariates inclusive 30 control probe PCs and K=9 latent dimensions instead of 6 cell mix adjustment as independent variables. Inflation due to genomic control is 0.765 and is 0.200 according to van Iterson's "bacon-method". [9]

Supplementary Table S27: Percent change in DNA-methylation by FFM unit (kg) for the top 50 probes from the reference free EWAS model (K=9).

Rank	CpG ID	CHR	Gene	UCSC REFGENE GROUP	RELATION TO UCSC CPG ISLAND	Position (bp)	% DNAm Change (SE) per FFM unit	*P*-value	Bon-ferroni	FDR
1	cg08692210	3	*WDR51A*	TSS200;TSS200;TSS200	Island	52188851	0.26 (0.05)	3.37E-07	1.45E-01	1.08E-01
2	cg19903596	5			Island	180527369	0.56 (0.11)	5.16E-07	2.22E-01	1.08E-01
3	cg07015354	15	*CA12*	TSS1500;TSS1500	S_Shore	63675552	0.31 (0.06)	7.52E-07	3.24E-01	1.08E-01
4	cg22789237	1				232684368	0.38 (0.08)	1.71E-06	7.39E-01	1.50E-01
5	cg21363348	7	*TMEM140*	TSS1500		134832221	-0.32 (0.07)	2.44E-06	1	1.50E-01
6	cg26884027	1	*PTPRU*	Body;Body;Body	Island	29586418	-0.08 (0.02)	2.54E-06	1	1.50E-01
7	cg25525825	6	*TRIM27*	Body	N_Shore	28889919	0.42 (0.09)	2.78E-06	1	1.50E-01
8	cg24999308	15	*NPTN*	1stExon;1stExon;1stExon;1stExon	Island	73925545	0.08 (0.02)	3.44E-06	1	1.50E-01
9	cg26721877	5	*STC2*	TSS1500	Island	172757074	0.28 (0.06)	3.75E-06	1	1.50E-01
10	cg22133562	12			S_Shore	115106488	0.23 (0.05)	3.80E-06	1	1.50E-01
11	cg00306523	8				94257369	0.30 (0.06)	4.60E-06	1	1.50E-01
12	cg01409343	17	*TMEM49*	Body		57915740	0.72 (0.16)	4.86E-06	1	1.50E-01
13	cg03125427	16	*CYBA*	TSS200	Island	88717482	-0.12 (0.03)	5.04E-06	1	1.50E-01
14	cg23470262	12	*GOLGA3*	Body		133383213	0.25 (0.05)	5.26E-06	1	1.50E-01
15	cg05911774	X	*SRPX2*	1stExon;5'UTR		99899345	0.29 (0.06)	5.52E-06	1	1.50E-01
16	cg23092576	1	*ECE1*	TSS1500	Island	21672253	0.05 (0.01)	5.66E-06	1	1.50E-01
17	cg03810768	2	*EDAR*	TSS200		109606018	0.51 (0.11)	5.97E-06	1	1.50E-01
18	cg01933946	1	*C1orf109*	Body	N_Shore	38155525	-0.05 (0.01)	6.28E-06	1	1.50E-01
19	cg06357843	22	*PANX2*	Body;Body;Body	Island	50616150	0.56 (0.12)	7.40E-06	1	1.68E-01
20	cg06318624	2				174913188	0.47 (0.10)	1.03E-05	1	1.97E-01
21	cg16520815	16	*MGRN1*	Body;Body;Body;Body	Island	4733181	0.57 (0.13)	1.04E-05	1	1.97E-01
22	cg13281171	6	*SYCP2L*	TSS200	N_Shore	10887003	0.12 (0.03)	1.18E-05	1	1.97E-01
23	cg05715492	7	*ARPC1B*	Body	S_Shore	98991138	0.72 (0.16)	1.18E-05	1	1.97E-01
24	cg05022368	8	*ADAM9*	Body;Body;Body;Body	S_Shelf	38858552	0.38 (0.09)	1.20E-05	1	1.97E-01
25	cg26794885	8				12908302	0.57 (0.13)	1.23E-05	1	1.97E-01
26	cg22590032	5	*FLT4*	Body;Body	S_Shore	180050565	0.29 (0.06)	1.24E-05	1	1.97E-01
27	cg15490703	19	*EFNA2*	Body	Island	1295624	0.24 (0.05)	1.30E-05	1	1.97E-01

28	cg27038634	8			N_Shore	49468341	0.51 (0.11)	1.30E-05	1	1.97E-01
29	cg01127586	X	*LOC92249*	TSS200		62780937	-0.50 (0.11)	1.35E-05	1	1.97E-01
30	cg21067465	11	*ME3*	Body;Body;Body		86304288	0.21 (0.05)	1.37E-05	1	1.97E-01
31	cg21620524	5	*SLC9A3*	Body	Island	500521	-0.28 (0.06)	1.49E-05	1	2.02E-01
32	cg04515533	1			N_Shore	231295786	0.30 (0.07)	1.50E-05	1	2.02E-01
33	cg03996020	7	*DNAJB6*	Body		157180124	0.65 (0.15)	1.64E-05	1	2.14E-01
34	cg13964068	9			Island	69197721	0.25 (0.06)	1.73E-05	1	2.19E-01
35	cg26072437	4	*CLCN3*	Body;Body		170581380	-0.36 (0.08)	1.84E-05	1	2.26E-01
36	cg26882525	5	*SLC36A3*	Body;Body		150677867	-2.71 (0.62)	1.90E-05	1	2.28E-01
37	cg03045133	6	*HCG2P7*	Body		29870056	-0.51 (0.12)	1.99E-05	1	2.32E-01
38	cg25378085	17	*EME1;MRPL27*	TSS200;Body;TSS200	Island	48450457	-0.09 (0.02)	2.17E-05	1	2.43E-01
39	cg16045677	6	*BTNL2*	Body		32362678	-0.26 (0.06)	2.20E-05	1	2.43E-01
40	cg14163502	9	*KCNT1*	Body		138619497	0.38 (0.09)	2.32E-05	1	2.45E-01
41	cg21483906	3	*CLASP2*	Body	N_Shore	33758870	0.27 (0.06)	2.38E-05	1	2.45E-01
42	cg22030032	19	*EGLN2*	Body;Body	N_Shelf	41312792	0.35 (0.08)	2.38E-05	1	2.45E-01
43	cg21515349	17	*NAGS;PYY*	TSS200;TSS200	Island	42082028	0.14 (0.03)	2.59E-05	1	2.55E-01
44	cg17278466	17	*USP36*	Body	Island	76798203	0.20 (0.05)	2.64E-05	1	2.55E-01
45	cg15661473	19	*NKPD1*	Body	Island	45656123	0.26 (0.06)	2.66E-05	1	2.55E-01
46	cg04546471	12	*RPL41*	TSS200;TSS200	N_Shore	56510299	-0.07 (0.02)	2.80E-05	1	2.63E-01
47	cg16170760	X	*LAGE3*	TSS1500	S_Shore	153708103	-0.27 (0.06)	2.90E-05	1	2.66E-01
48	cg23969559	1	*ETV3*	TSS1500;TSS1500	S_Shore	157108676	-0.07 (0.02)	3.00E-05	1	2.69E-01
49	cg19358373	12			S_Shore	133414571	0.46 (0.11)	3.08E-05	1	2.71E-01
50	cg12121643	3	*DGKG*	Body;Body;Body	Island	185911796	0.40 (0.09)	3.22E-05	1	2.78E-01

Genes in brackets indicate manual annotation for closest genes using the UCSC genome browser on human GRCh37/hg19 assembly. The record highlighted in red indicates the last methylation site significantly associated with the outcome acccording to Bonferroni correction and the blue highlighted record the last signficant according to false-discovery rate.[5] Here no methylation site is significantly asscociated neither by Bonferroni nor by FDR criteria. The estimates and *P*-values are based on the reference free EWAS model of Houseman [6,7] with DNAm as outcome and phenotype, all covariates inclusive 30 control probe PCs and K=9 latent dimensions instead of 6 cell mix adjustment as independent variables. Inflation due to genomic control is 0.825 and is 0.203 according to van Iterson's "bacon-method". [9]

Supplementary Table S28: Percent change in DNA-methylation by FFMI unit (kg/m²) for the top 50 probes from the reference free EWAS model (K=9).

Rank	CpG ID	CHR	Gene	UCSC REFGENE GROUP	RELATION TO UCSC CPG ISLAND	Position (bp)	% DNAm change (SE) per FFMI unit	*P*-value	Bon-ferroni	FDR
1	cg21165061	15	*DISP2*	Body	Island	40651101	0.88 (0.16)	5.52E-08	2.38E-02	2.38E-02
2	cg05715492	7	*ARPC1B*	Body	S_Shore	98991138	1.66 (0.31)	1.93E-07	8.34E-02	2.98E-02
3	cg06437396	6			N_Shore	108437013	0.53 (0.10)	2.07E-07	8.93E-02	2.98E-02
4	cg04010122	2	*SOS1*	Body	N_Shore	39346883	0.44 (0.09)	5.93E-07	2.56E-01	5.49E-02
5	cg24586432	2	*SCLY*	TSS1500	Island	238969253	0.13 (0.03)	6.36E-07	2.74E-01	5.49E-02
6	cg08692210	3	*WDR51A*	TSS200;TSS200;TSS200	Island	52188851	0.50 (0.10)	1.04E-06	4.48E-01	7.47E-02
7	cg26867987	6	*COL11A2*	3'UTR;3'UTR;3'UTR	S_Shore	33131450	-0.68 (0.14)	1.58E-06	6.81E-01	8.76E-02
8	cg02518775	11	*CNIH2*	Body	S_Shore	66046770	0.72 (0.15)	1.62E-06	7.01E-01	8.76E-02
9	cg22798261	19	*THEG*	TSS1500;TSS1500	S_Shore	376653	-2.08 (0.43)	2.02E-06	8.72E-01	9.18E-02
10	cg04764597	10	*C10orf107*	Body		63510947	0.35 (0.07)	2.13E-06	9.18E-01	9.18E-02
11	cg03091512	2	*RHBDD1*	5'UTR;5'UTR	Island	227701050	-0.17 (0.04)	3.08E-06	1	1.14E-01
12	cg01178624	11	*KCNK7*	3'UTR;3'UTR;3'UTR;3'UTR	Island	65360327	2.13 (0.45)	3.22E-06	1	1.14E-01
13	cg17935297	19	*CILP2*	Body	Island	19651968	0.28 (0.06)	3.64E-06	1	1.14E-01
14	cg10428938	7	*ARPC1B*	Body	Island	98990837	1.71 (0.36)	3.71E-06	1	1.14E-01
15	cg04219544	17	*KRT24*	TSS200		38860042	-0.84 (0.18)	4.01E-06	1	1.15E-01
16	cg17603909	11	*OR52K1*	TSS1500		4509357	0.61 (0.13)	5.09E-06	1	1.35E-01
17	cg03545246	1			Island	227976522	0.24 (0.05)	5.34E-06	1	1.35E-01
18	cg10119082	7	*ARPC1B*	Body	Island	98990626	0.90 (0.20)	7.24E-06	1	1.73E-01
19	cg11252801	2	*LRRTM4*	5'UTR;5'UTR		77748917	0.70 (0.15)	7.62E-06	1	1.73E-01
20	cg26882525	5	*SLC36A3*	Body;Body		150677867	-5.73 (1.27)	8.58E-06	1	1.79E-01
21	cg14759565	11			Island	65360123	1.89 (0.42)	8.79E-06	1	1.79E-01
22	cg00271204	5			N_Shore	139142898	0.62 (0.14)	9.12E-06	1	1.79E-01
23	cg06552618	12	*SMUG1*	5'UTR		54582532	-0.22 (0.05)	1.02E-05	1	1.92E-01
24	cg12379720	22	*TRIOBP*	TSS200		38092989	0.40 (0.09)	1.09E-05	1	1.93E-01
25	cg01976623	14	*PRIMA1*	TSS1500	S_Shore	94255901	0.93 (0.21)	1.12E-05	1	1.93E-01
26	cg25525825	6	*TRIM27*	Body	N_Shore	28889919	0.81 (0.18)	1.31E-05	1	2.17E-01
27	cg26401512	1	*ZNF643*	Body		40922773	-0.65 (0.15)	1.43E-05	1	2.28E-01

28	cg04267436	14	*ZDHHC22*	TSS200	Island	77608303	0.47 (0.11)	1.55E-05	1	2.39E-01
29	cg24398379	7	*LOC650226*	TSS200	S_Shore	56516076	-1.33 (0.30)	1.76E-05	1	2.53E-01
30	cg25941299	7			S_Shore	113727916	1.16 (0.27)	1.76E-05	1	2.53E-01
31	cg24999308	15	*NPTN*	1stExon;1stExon;1stExon;1stExon	Island	73925545	0.16 (0.04)	1.83E-05	1	2.53E-01
32	cg06965803	1	*PLEKHG5*	Body;Body;Body;Body;Body	Island	6531717	0.99 (0.23)	1.88E-05	1	2.53E-01
33	cg00250743	10	*FGFR2*	Body;Body;Body;Body;Body; Body;Body;Body;Body		123264130	-0.66 (0.15)	1.93E-05	1	2.53E-01
34	cg07764575	19	*CATSPERG*	Body	N_Shore	38850992	-0.81 (0.19)	2.21E-05	1	2.80E-01
35	cg05709879	5			N_Shelf	141067731	-1.08 (0.25)	2.33E-05	1	2.87E-01
36	cg10866060	12				83592588	1.30 (0.30)	2.51E-05	1	3.00E-01
37	cg14124917	2	*GRHL1*	TSS1500	Island	10090974	0.34 (0.08)	2.62E-05	1	3.05E-01
38	cg15026574	3	*ST6GAL1*	5'UTR;5'UTR		186683429	-0.55 (0.13)	2.86E-05	1	3.24E-01
39	cg16810626	8	*GATA4*	Body		11594003	1.34 (0.32)	3.09E-05	1	3.34E-01
40	cg13654525	11	*KCNK7*	Body;3'UTR;3'UTR;3'UTR	S_Shore	65360509	1.19 (0.28)	3.11E-05	1	3.34E-01
41	cg15059562	7			Island	65235696	0.48 (0.11)	3.17E-05	1	3.34E-01
42	cg03810768	2	*EDAR*	TSS200		109606018	0.92 (0.22)	3.39E-05	1	3.37E-01
43	cg12321193	13	*EDNRB*	TSS1500		78550384	-0.62 (0.15)	3.44E-05	1	3.37E-01
44	cg21532325	7	*WDR86*	TSS1500	Island	151107400	-0.23 (0.05)	3.48E-05	1	3.37E-01
45	cg12610207	12	*TBX5*	Body;Body;Body;Body	Island	114833950	0.14 (0.03)	3.52E-05	1	3.37E-01
46	cg08144588	3	*CNTN4*	Body;TSS1500;Body		3080327	2.23 (0.53)	3.84E-05	1	3.44E-01
47	cg05911774	X	*SRPX2*	1stExon;5'UTR		99899345	0.57 (0.14)	3.99E-05	1	3.44E-01
48	cg09245901	6				132075947	1.18 (0.28)	4.02E-05	1	3.44E-01
49	cg16570885	3	*IGF2BP2*	Body;Body		185476452	-5.13 (1.23)	4.08E-05	1	3.44E-01
50	cg15466176	12				114781410	0.67 (0.16)	4.14E-05	1	3.44E-01

Genes in brackets indicate manual annotation for closest genes using the UCSC genome browser on human GRCh37/hg19 assembly. The record highlighted in red indicates the last methylation site significantly associated with the outcome acccording to Bonferroni correction and the blue highlighted record the last signficant according to false-discovery rate.[5] The estimates and *P*-values are based on the reference free EWAS model of Houseman [6,7] with DNAm as outcome and phenotype, all covariates inclusive 30 control probe PCs and K=9 latent dimensions instead of 6 cell mix adjustment as independent variables. Inflation due to genomic control[8] is 0.743 and is 0.198 according to van Iterson's "bacon-method". [9]

Supplementary Table S29: Change in BMI (kg/m^2) per percent change in DNA-methylation adj. for matenal smoking during pregnancy (top 50 probes).

Rank	CpG ID	CHR	Gene	UCSC REFGENE GROUP	RELATION TO UCSC CPG ISLAND	Position (bp)	Change (SE) in BMI per % change in DNAm	*P*-value	Bon-ferroni	FDR
1	cg13850887	2	SNED1	Body	S_Shore	241993303	-0.21 (0.04)	3.68E-08	1.59E-02	1.59E-02
2	cg01706498	3	KLHL6	Body		183260375	0.19 (0.03)	8.68E-08	3.74E-02	1.87E-02
3	cg26401512	1	ZNF643	Body		40922773	-0.20 (0.04)	1.50E-07	6.46E-02	2.15E-02
4	cg17935297	19	CILP2	Body	Island	19651968	0.41 (0.08)	3.18E-07	1.37E-01	2.81E-02
5	cg15026574	3	ST6GAL1	5'UTR;5'UTR		186683429	-0.21 (0.04)	3.26E-07	1.41E-01	2.81E-02
6	cg26867987	6	COL11A2	3'UTR;3'UTR;3'UTR	S_Shore	33131450	-0.18 (0.04)	4.97E-07	2.14E-01	2.87E-02
7	cg21525627	12	(ZDHHC17)			77149647	0.24 (0.05)	5.31E-07	2.29E-01	2.87E-02
8	cg21126338	13	FARP1	Body		99084698	0.32 (0.06)	5.49E-07	2.37E-01	2.87E-02
9	cg17810765	2	ANO7	TSS200;TSS200		242127836	0.14 (0.03)	5.98E-07	2.58E-01	2.87E-02
10	cg18704658	17			N_Shelf	80300165	0.38 (0.08)	7.28E-07	3.14E-01	3.00E-02
11	cg26618041	19	CILP2	Body	Island	19651867	0.19 (0.04)	7.74E-07	3.34E-01	3.00E-02
12	cg06594770	22	TRIOBP	TSS200		38092974	0.44 (0.09)	8.35E-07	3.60E-01	3.00E-02
13	cg17989572	17	RAB5C	5'UTR;5'UTR	N_Shore	40306700	-0.51 (0.10)	9.29E-07	4.01E-01	3.08E-02
14	cg08074767	6	(MLLT4)			168113741	-0.12 (0.02)	1.07E-06	4.60E-01	3.19E-02
15	cg27285599	17	TBCD;ZNF750	Body;TSS200		80798023	0.33 (0.07)	1.18E-06	5.08E-01	3.19E-02
16	cg06369443	1	KCNQ4	Body;Body	N_Shore	41303048	0.34 (0.07)	1.33E-06	5.73E-01	3.19E-02
17	cg14518658	22	(CYTH4-ELFN2)		N_Shelf	37726676	0.11 (0.02)	1.40E-06	6.03E-01	3.19E-02
18	cg06322432	10			N_Shore	112289853	0.13 (0.03)	1.49E-06	6.43E-01	3.19E-02
19	cg24751284	14	APEX1	Body;Body;Body	S_Shore	20925551	0.23 (0.05)	1.50E-06	6.49E-01	3.19E-02
20	cg26917480	17	ADAP2	TSS1500	N_Shore	29247893	0.25 (0.05)	1.61E-06	6.94E-01	3.19E-02
21	cg26995653	2	(LINC01115 (TMEM18))			858038	0.11 (0.02)	1.62E-06	7.01E-01	3.19E-02
22	cg14795409	3				128267611	-0.20 (0.04)	1.70E-06	7.33E-01	3.19E-02
23	cg14391016	6	CCR6	TSS1500		167523797	0.23 (0.05)	1.91E-06	8.23E-01	3.19E-02
24	cg06437396	6	OSTM1		N_Shore	108437013	0.20 (0.04)	1.97E-06	8.50E-01	3.19E-02
25	cg15941159	22	GRAMD4	3'UTR	S_Shore	47075270	0.23 (0.05)	1.99E-06	8.58E-01	3.19E-02

26	cg14950834	19	SPTBN4;BLVRB	TSS1500;TSS200	Island	40971830	-0.55 (0.11)	2.00E-06	8.63E-01	3.19E-02
27	cg13298389	7				49710144	0.58 (0.12)	2.12E-06	9.14E-01	3.19E-02
28	cg01257171	5			S_Shore	172656713	0.41 (0.08)	2.12E-06	9.14E-01	3.19E-02
29	cg14401837	7	NPSR1	TSS1500;TSS1500		34697493	-0.24 (0.05)	2.15E-06	9.26E-01	3.19E-02
30	cg23416307	4	GAK	Body		858299	0.63 (0.13)	2.24E-06	9.65E-01	3.22E-02
31	cg21332304	17	NPEPPS	Body		45699260	-0.35 (0.07)	2.31E-06	9.98E-01	3.22E-02
32	cg10802005	12				121404850	0.30 (0.06)	2.52E-06	1.00E+00	3.39E-02
33	cg06217450	3			N_Shore	142441654	0.44 (0.09)	2.84E-06	1.00E+00	3.52E-02
34	cg02682525	11	ANKK1	TSS1500	N_Shore	113258277	0.14 (0.03)	2.85E-06	1.00E+00	3.52E-02
35	cg17287326	10	AVPI1	5'UTR;1stExon	Island	99446966	0.33 (0.07)	2.89E-06	1.00E+00	3.52E-02
36	cg06376715	1	TP73	Body;Body;Body;Body	Island	3634918	0.10 (0.02)	3.05E-06	1.00E+00	3.52E-02
37	cg10861407	9	FSD1L	TSS1500;TSS1500;TSS1500	N_Shore	108209278	-0.25 (0.05)	3.20E-06	1.00E+00	3.52E-02
38	cg02381820	2			Island	121493896	0.45 (0.10)	3.25E-06	1.00E+00	3.52E-02
39	cg00384539	8	PRDM14	TSS200	Island	70983567	0.29 (0.06)	3.45E-06	1.00E+00	3.52E-02
40	cg00193021	4	SNCA	5'UTR;1stExon;5'UTR;1stExon;1stExon;5'UTR;5'UTR	Island	90758120	-0.27 (0.06)	3.49E-06	1.00E+00	3.52E-02
41	cg03012642	2				21346892	-0.19 (0.04)	3.51E-06	1.00E+00	3.52E-02
42	cg11245333	1	MOSC1	TSS200	N_Shore	220959931	0.24 (0.05)	3.56E-06	1.00E+00	3.52E-02
43	cg08568550	11	C11orf16	TSS200		8954570	0.25 (0.05)	3.57E-06	1.00E+00	3.52E-02
44	ch.8.1234861F	8	XKR4	Body		56321773	-0.82 (0.17)	3.65E-06	1.00E+00	3.52E-02
45	cg13345817	15				101695660	0.28 (0.06)	3.79E-06	1.00E+00	3.52E-02
46	cg04813904	5	CD180	Body		66488838	0.13 (0.03)	3.84E-06	1.00E+00	3.52E-02
47	cg13274254	2	GULP1	5'UTR;1stExon	Island	189157566	0.32 (0.07)	3.85E-06	1.00E+00	3.52E-02
48	cg24332767	3	C3orf70	TSS1500	Island	184871524	0.15 (0.03)	3.95E-06	1.00E+00	3.52E-02
49	cg25048701	11	FOLR1	5'UTR;TSS1500;5'UTR;5'UTR		71902284	0.43 (0.09)	4.00E-06	1.00E+00	3.52E-02
50	cg15650694	5	SFRS12	TSS200;TSS1500	Island	65439990	0.29 (0.06)	4.37E-06	1.00E+00	3.64E-02

Genes in brackets indicate manual annotation for closest genes using the UCSC genome browser on human GRCh37/hg19 assembly. The record highlighted in red indicates the last methylation site significantly associated with the outcome acccording to Bonferroni correction and the blue highlighted record the last signficant according to false-discovery rate.[5] Cell type heterogeneity is accounted for by adjustment for 6 WBC types according to Houseman[10] (see methods section of main manuscript). EWAS model is same as main reference-based EWAS model (see Supplementary Tables S1-S6) but further adjusted for indicator coded variable "Any maternal smoking during pregnancy (y/n)".

Supplementary Table S30: Change in WHO stand. BMI per percent change in DNA-methylation adj. for maternal smoking during pregnancy (top 50 probes).

Rank	CpG ID	CHR	Gene	UCSC REFGENE GROUP	RELATION TO UCSC CPG ISLAND	Position (bp)	Change (SE) in ZBMI per % change in DNAm	*P*-value	Bon-ferroni	FDR
1	cg01706498	3	*KLHL6*	Body		183260375	0.11 (0.02)	1.90E-07	8.20E-02	4.24E-02
2	cg21126338	13	*FARP1*	Body		99084698	0.20 (0.04)	1.98E-07	8.52E-02	4.24E-02
3	cg13850887	2	*SNED1*	Body	S_Shore	241993303	-0.12 (0.02)	2.95E-07	1.27E-01	4.24E-02
4	cg17935297	19	*CILP2*	Body	Island	19651968	0.24 (0.05)	4.37E-07	1.88E-01	4.71E-02
5	cg21525627	12	*(ZDHHC17)*			77149647	0.14 (0.03)	5.80E-07	2.50E-01	5.00E-02
6	cg24751284	14	*APEX1*	Body;Body;Body	S_Shore	20925551	0.14 (0.03)	9.66E-07	4.16E-01	6.03E-02
7	cg27285599	17	*TBCD;ZNF750*	Body;TSS200		80798023	0.20 (0.04)	1.07E-06	4.60E-01	6.03E-02
8	cg26618041	19	*CILP2*	Body	Island	19651867	0.11 (0.02)	1.23E-06	5.29E-01	6.03E-02
9	cg23416307	4	*GAK*	Body		858299	0.39 (0.08)	1.39E-06	6.01E-01	6.03E-02
10	cg06369443	1	*KCNQ4*	Body;Body	N_Shore	41303048	0.20 (0.04)	1.76E-06	7.60E-01	6.03E-02
11	cg08074767	6	*(MLLT4)*			168113741	-0.07 (0.01)	2.24E-06	9.65E-01	6.03E-02
12	cg15660740	17			N_Shore	81023473	-0.24 (0.05)	2.69E-06	1.00E+00	6.03E-02
13	cg17989572	17	*RAB5C*	5'UTR;5'UTR	N_Shore	40306700	-0.30 (0.06)	2.71E-06	1.00E+00	6.03E-02
14	cg18704658	17			N_Shelf	80300165	0.22 (0.05)	3.06E-06	1.00E+00	6.03E-02
15	cg04813904	5	*CD180*	Body		66488838	0.08 (0.02)	3.22E-06	1.00E+00	6.03E-02
16	cg26917480	17	*ADAP2*	TSS1500	N_Shore	29247893	0.15 (0.03)	3.24E-06	1.00E+00	6.03E-02
17	cg26995653	2	*(LINC01115 (TMEM18))*			858038	0.07 (0.01)	3.34E-06	1.00E+00	6.03E-02
18	cg11504355	1	*KIRREL*	3'UTR		158065595	0.09 (0.02)	3.55E-06	1.00E+00	6.03E-02
19	cg14391016	6	*CCR6*	TSS1500		167523797	0.14 (0.03)	3.55E-06	1.00E+00	6.03E-02
20	cg14950834	19	*SPTBN4;BLVRB*	TSS1500;TSS200	Island	40971830	-0.33 (0.07)	3.55E-06	1.00E+00	6.03E-02
21	cg06437396	6	*(OSTM1)*		N_Shore	108437013	0.12 (0.03)	3.65E-06	1.00E+00	6.03E-02
22	cg26401512	1	*ZNF643*	Body		40922773	-0.11 (0.02)	3.69E-06	1.00E+00	6.03E-02
23	cg04979599	5	*AFAP1L1*	TSS1500;TSS1500	N_Shore	148650899	0.13 (0.03)	3.78E-06	1.00E+00	6.03E-02
24	cg17810765	2	*ANO7;ANO7*	TSS200;TSS200		242127836	0.08 (0.02)	3.93E-06	1.00E+00	6.03E-02
25	cg14401837	7	*NPSR1*	TSS1500;TSS1500		34697493	-0.14 (0.03)	4.21E-06	1.00E+00	6.03E-02

26	cg14518658	22	*(CYTH4-ELFN2)*		N_Shelf	37726676	0.06 (0.01)	4.27E-06	1.00E+00	6.03E-02
27	cg13298389	7				49710144	0.34 (0.07)	4.37E-06	1.00E+00	6.03E-02
28	cg25048701	11	*FOLR1*	5'UTR;TSS1500;5'UTR;5'UTR		71902284	0.26 (0.06)	4.40E-06	1.00E+00	6.03E-02
29	cg06322432	10			N_Shore	112289853	0.07 (0.02)	4.65E-06	1.00E+00	6.03E-02
30	cg10802005	12				121404850	0.18 (0.04)	4.85E-06	1.00E+00	6.03E-02
31	cg17287326	10	*AVPI1*	5'UTR;1stExon	Island	99446966	0.20 (0.04)	5.12E-06	1.00E+00	6.03E-02
32	ch.8.1234861F	8	*XKR4*	Body		56321773	-0.49 (0.11)	5.32E-06	1.00E+00	6.03E-02
33	cg13345817	15				101695660	0.17 (0.04)	5.54E-06	1.00E+00	6.03E-02
34	cg13274254	2	*GULP1*	5'UTR;1stExon	Island	189157566	0.19 (0.04)	5.57E-06	1.00E+00	6.03E-02
35	cg15026574	3	*ST6GAL1*	5'UTR;5'UTR		186683429	-0.11 (0.02)	5.67E-06	1.00E+00	6.03E-02
36	cg00384539	8	*PRDM14*	TSS200	Island	70983567	0.17 (0.04)	5.72E-06	1.00E+00	6.03E-02
37	cg10861407	9	*FSD1L*	TSS1500;TSS1500;TSS1500	N_Shore	108209278	-0.15 (0.03)	6.02E-06	1.00E+00	6.03E-02
38	cg14524775	1	*ARHGEF2*	Body;Body;Body	Island	155920832	0.42 (0.09)	6.28E-06	1.00E+00	6.03E-02
39	cg14795409	3				128267611	-0.12 (0.03)	6.35E-06	1.00E+00	6.03E-02
40	cg11245333	1	*MOSC1*	TSS200	N_Shore	220959931	0.14 (0.03)	6.39E-06	1.00E+00	6.03E-02
41	cg26666886	16	*ANKRD11*	TSS1500	S_Shore	89558024	-0.19 (0.04)	6.45E-06	1.00E+00	6.03E-02
42	cg08568550	11	*C11orf16*	TSS200		8954570	0.15 (0.03)	6.46E-06	1.00E+00	6.03E-02
43	cg24067118	10			N_Shore	102806933	0.05 (0.01)	6.46E-06	1.00E+00	6.03E-02
44	cg15941159	22	*GRAMD4*	3'UTR	S_Shore	47075270	0.13 (0.03)	6.86E-06	1.00E+00	6.03E-02
45	cg22503047	6	*BAT2*	Body	S_Shore	31601411	-0.19 (0.04)	6.88E-06	1.00E+00	6.03E-02
46	cg25371332	2				10688317	0.11 (0.02)	6.94E-06	1.00E+00	6.03E-02
47	cg26879644	15				92238013	0.17 (0.04)	6.96E-06	1.00E+00	6.03E-02
48	cg06376715	1	*TP73*	Body;Body;Body;Body	Island	3634918	0.06 (0.01)	7.03E-06	1.00E+00	6.03E-02
49	cg27247382	2	*PLEKHM3*	3'UTR		208689681	0.32 (0.07)	7.04E-06	1.00E+00	6.03E-02
50	cg09204618	22	*TSSK2*	TSS1500	N_Shelf	19117090	0.18 (0.04)	7.37E-06	1.00E+00	6.03E-02

Genes in brackets indicate manual annotation for closest genes using the UCSC genome browser on human GRCh37/hg19 assembly. The record highlighted in red indicates the last methylation site significantly associated with the outcome acccording to Bonferroni correction and the blue highlighted record the last signficant according to false-discovery rate.[5] Cell type heterogeneity is accounted for by adjustment for 6 WBC types according to Houseman[10] (see methods section of main manuscript). EWAS model is same as main reference-based EWAS model (see Supplementary Tables S1-S6) but further adjusted for indicator coded variable “Any maternal smoking during pregnancy (y/n)”.

Supplementary Table S31: Change in fat mass (kg) per percent change in DNA-methylation adj. for maternal smoking during pregnancy (top 50 probes).

Rank	CpG ID	CHR	Gene	UCSC REFGENE GROUP	RELATION TO UCSC CPG ISLAND	Position (bp)	Change (SE) in FM per % change in DNAm	P-value	Bon-ferroni	FDR
1	cg13850887	2	*SNED1*	Body	S_Shore	241993303	-0.20 (0.03)	1.38E-10	5.93E-05	5.93E-05
2	cg08692210	3	*WDR51A*	TSS200;TSS200;TSS200	Island	52188851	0.17 (0.03)	7.20E-08	3.11E-02	1.11E-02
3	cg00384539	8	*PRDM14*	TSS200	Island	70983567	0.27 (0.05)	8.91E-08	3.84E-02	1.11E-02
4	cg15026574	3	*ST6GAL1*	5'UTR;5'UTR		186683429	-0.18 (0.03)	1.03E-07	4.43E-02	1.11E-02
5	cg14518658	22	*(CYTH4-ELFN2)*		N_Shelf	37726676	0.10 (0.02)	1.99E-07	8.57E-02	1.23E-02
6	cg04894009	8	*PRKDC*	Body;Body		48695125	0.31 (0.06)	2.22E-07	9.58E-02	1.23E-02
7	cg26627956	2	*CFLAR*	Body;Body;Body		202004766	-0.19 (0.04)	2.23E-07	9.62E-02	1.23E-02
8	cg26401512	1	*ZNF643*	Body		40922773	-0.16 (0.03)	2.30E-07	9.93E-02	1.23E-02
9	cg06594770	22	*TRIOBP*	TSS200		38092974	0.36 (0.07)	2.98E-07	1.28E-01	1.23E-02
10	cg04010122	2	*SOS1*	Body	N_Shore	39346883	0.16 (0.03)	3.34E-07	1.44E-01	1.23E-02
11	cg13641993	9	*FBXO10*	5'UTR		37559166	0.22 (0.04)	3.43E-07	1.48E-01	1.23E-02
12	cg01462036	16	*TBL3*	Body	S_Shore	2025868	-0.13 (0.03)	3.94E-07	1.70E-01	1.23E-02
13	cg27038634	8	*(LOC101929268)*		N_Shore	49468341	0.09 (0.02)	4.26E-07	1.84E-01	1.23E-02
14	cg01257171	5			S_Shore	172656713	0.35 (0.07)	4.26E-07	1.84E-01	1.23E-02
15	cg02381820	2			Island	121493896	0.39 (0.08)	4.26E-07	1.84E-01	1.23E-02
16	cg21525627	12	*(ZDHHC17)*			77149647	0.20 (0.04)	5.28E-07	2.28E-01	1.42E-02
17	cg01706498	3	*KLHL6*	Body		183260375	0.14 (0.03)	6.12E-07	2.64E-01	1.55E-02
18	cg15941159	22	*GRAMD4*	3'UTR	S_Shore	47075270	0.19 (0.04)	6.98E-07	3.01E-01	1.67E-02
19	cg13153009	8	*ZC3H3*	Body	N_Shelf	144561626	-0.60 (0.12)	7.65E-07	3.30E-01	1.74E-02
20	cg14401837	7	*NPSR1*	TSS1500;TSS1500		34697493	-0.20 (0.04)	1.04E-06	4.49E-01	2.16E-02
21	cg14795409	3				128267611	-0.17 (0.03)	1.05E-06	4.54E-01	2.16E-02
22	cg26867987	6	*COL11A2*	3'UTR;3'UTR;3'UTR	S_Shore	33131450	-0.14 (0.03)	1.17E-06	5.05E-01	2.30E-02
23	cg06376715	1	*TP73*	Body;Body;Body;Body	Island	3634918	0.08 (0.02)	1.24E-06	5.33E-01	2.32E-02
24	cg08074767	6	*(MLLT4)*			168113741	-0.09 (0.02)	1.62E-06	6.98E-01	2.91E-02
25	cg12717591	10	*SFMBT2*	3'UTR		7205138	0.36 (0.07)	1.69E-06	7.27E-01	2.91E-02

26	cg14240060	10				130830949	0.23 (0.05)	1.87E-06	8.07E-01	3.10E-02
27	cg24332767	3	*C3orf70*	TSS1500	Island	184871524	0.12 (0.03)	1.99E-06	8.58E-01	3.18E-02
28	cg10822034	11				10529596	-0.19 (0.04)	2.18E-06	9.40E-01	3.27E-02
29	cg13298389	7				49710144	0.47 (0.10)	2.20E-06	9.47E-01	3.27E-02
30	cg21126338	13	*FARP1*	Body		99084698	0.24 (0.05)	2.35E-06	1.00E+00	3.28E-02
31	cg17730176	17	*TIAF1;MYO18A*	TSS1500;Body;Body	N_Shelf	27404126	0.26 (0.05)	2.46E-06	1.00E+00	3.28E-02
32	cg13008301	20	*CSTF1*	Body;Body;Body	Island	54978749	-0.09 (0.02)	2.52E-06	1.00E+00	3.28E-02
33	cg03174228	9	*TTLL11*	Body	N_Shore	124658583	0.07 (0.01)	2.55E-06	1.00E+00	3.28E-02
34	cg02431597	1	*CACNA1E*	TSS1500	N_Shore	181452137	0.12 (0.03)	2.78E-06	1.00E+00	3.28E-02
35	cg06322432	10			N_Shore	112289853	0.10 (0.02)	2.86E-06	1.00E+00	3.28E-02
36	cg00193021	4	*SNCA*	5'UTR;1stExon;5'UTR;1stExon;1stExon;5'UTR;5'UTR	Island	90758120	-0.22 (0.05)	2.89E-06	1.00E+00	3.28E-02
37	cg14699734	19			S_Shore	5806164	0.09 (0.02)	2.92E-06	1.00E+00	3.28E-02
38	cg20380470	10			N_Shore	131987945	0.38 (0.08)	2.95E-06	1.00E+00	3.28E-02
39	cg12076692	1	*GNB1*	Body		1718852	0.31 (0.06)	3.34E-06	1.00E+00	3.28E-02
40	cg26002437	10	*C10orf10;RASSF4*	Body;Body	S_Shelf	45473231	0.21 (0.04)	3.37E-06	1.00E+00	3.28E-02
41	cg17810765	2	*ANO7*	TSS200;TSS200		242127836	0.11 (0.02)	3.38E-06	1.00E+00	3.28E-02
42	cg06526681	6	*TSPYL4*	3'UTR;1stExon	N_Shore	116573109	-0.15 (0.03)	3.39E-06	1.00E+00	3.28E-02
43	cg22030032	19	*EGLN2*	Body;Body	N_Shelf	41312792	0.11 (0.02)	3.44E-06	1.00E+00	3.28E-02
44	cg17989572	17	*RAB5C*	5'UTR;5'UTR	N_Shore	40306700	-0.39 (0.08)	3.46E-06	1.00E+00	3.28E-02
45	cg10413089	15	*CYFIP1*	5'UTR		22921426	0.19 (0.04)	3.58E-06	1.00E+00	3.28E-02
46	cg18704658	17			N_Shelf	80300165	0.29 (0.06)	3.71E-06	1.00E+00	3.28E-02
47	cg02942594	19	*LOC148189*	Body	Island	28284741	-0.31 (0.07)	3.81E-06	1.00E+00	3.28E-02
48	cg26917480	17	*ADAP2*	TSS1500	N_Shore	29247893	0.19 (0.04)	3.87E-06	1.00E+00	3.28E-02
49	cg18051668	17			S_Shelf	55126941	0.26 (0.06)	3.88E-06	1.00E+00	3.28E-02
50	cg00145141	17	*GGT6*	TSS200;TSS200		4464022	-0.18 (0.04)	3.99E-06	1.00E+00	3.28E-02

Genes in brackets indicate manual annotation for closest genes using the UCSC genome browser on human GRCh37/hg19 assembly. The record highlighted in red indicates the last methylation site significantly associated with the outcome acccording to Bonferroni correction and the blue highlighted record the last signficant according to false-discovery rate.[5] Cell type heterogeneity is accounted for by adjustment for 6 WBC types according to Houseman[10] (see methods section of main manuscript). EWAS model is same as main reference-based EWAS model (see Supplementary Tables S1-S6) but further adjusted for indicator coded variable “Any maternal smoking during pregnancy (y/n)”.

Supplementary Table S32: Change in fat mass index (kg/m²) per percent change in DNA-methylation adj. for maternal smoking in pregnancy (top 50 probes).

Rank	CpG ID	CHR	Gene	UCSC REFGENE GROUP	RELATION TO UCSC CPG ISLAND	Position (bp)	Change (SE) in FMI per % change in DNAm	*P*-value	Bon-ferroni	FDR
1	cg13850887	2	*SNED1*	Body	S_Shore	241993303	-0.14 (0.02)	1.12E-11	4.85E-06	4.85E-06
2	cg26401512	1	*ZNF643*	Body		40922773	-0.12 (0.02)	3.17E-08	1.37E-02	3.96E-03
3	cg15026574	3	*ST6GAL1*	5'UTR;5'UTR		186683429	-0.12 (0.02)	3.34E-08	1.44E-02	3.96E-03
4	cg04010122	2	*SOS1*	Body	N_Shore	39346883	0.12 (0.02)	3.67E-08	1.58E-02	3.96E-03
5	cg26627956	2	*CFLAR*	Body;Body;Body		202004766	-0.14 (0.02)	8.65E-08	3.73E-02	7.46E-03
6	cg06594770	22	*TRIOBP*	TSS200		38092974	0.25 (0.05)	1.45E-07	6.25E-02	1.04E-02
7	cg14401837	7	*NPSR1*	TSS1500;TSS1500		34697493	-0.14 (0.03)	3.06E-07	1.32E-01	1.76E-02
8	cg08692210	3	*WDR51A*	TSS200;TSS200;TSS200	Island	52188851	0.11 (0.02)	3.58E-07	1.55E-01	1.76E-02
9	cg26867987	6	*COL11A2*	3'UTR;3'UTR;3'UTR	S_Shore	33131450	-0.10 (0.02)	3.81E-07	1.64E-01	1.76E-02
10	cg14518658	22	*(CYTH4-ELFN2)*		N_Shelf	37726676	0.06 (0.01)	4.35E-07	1.88E-01	1.76E-02
11	cg04894009	8	*PRKDC*	Body;Body		48695125	0.21 (0.04)	4.50E-07	1.94E-01	1.76E-02
12	cg00384539	8	*PRDM14*	TSS200	Island	70983567	0.17 (0.03)	4.99E-07	2.15E-01	1.79E-02
13	cg01706498	3	*KLHL6*	Body		183260375	0.10 (0.02)	5.68E-07	2.45E-01	1.88E-02
14	cg13641993	9	*FBXO10*	5'UTR		37559166	0.15 (0.03)	9.94E-07	4.29E-01	3.06E-02
15	cg15941159	22	*GRAMD4*	3'UTR	S_Shore	47075270	0.13 (0.03)	1.08E-06	4.68E-01	3.12E-02
16	cg01257171	5			S_Shore	172656713	0.23 (0.05)	1.19E-06	5.14E-01	3.21E-02
17	cg01462036	16	*TBL3*	Body	S_Shore	2025868	-0.09 (0.02)	1.58E-06	6.82E-01	3.84E-02
18	cg24093300	7	*PTPRN2*	Body;Body;Body		157543805	-0.11 (0.02)	1.60E-06	6.92E-01	3.84E-02
19	cg12717591	10	*SFMBT2*	3'UTR		7205138	0.24 (0.05)	1.84E-06	7.92E-01	4.17E-02
20	cg10515155	17			Island	81052671	-0.08 (0.02)	2.12E-06	9.15E-01	4.57E-02
21	cg02682525	11	*ANKK1*	TSS1500	N_Shore	113258277	0.08 (0.02)	2.23E-06	9.62E-01	4.58E-02
22	cg02381820	2			Island	121493896	0.25 (0.05)	2.36E-06	1.00E+00	4.63E-02
23	cg21525627	12	*(ZDHHC17)*			77149647	0.13 (0.03)	2.76E-06	1.00E+00	4.97E-02
24	cg10413089	15	*CYFIP1*	5'UTR		22921426	0.13 (0.03)	2.77E-06	1.00E+00	4.97E-02
25	cg02431597	1	*CACNA1E*	TSS1500	N_Shore	181452137	0.08 (0.02)	3.06E-06	1.00E+00	5.21E-02
26	cg17810765	2	*ANO7*	TSS200;TSS200		242127836	0.07 (0.02)	3.14E-06	1.00E+00	5.21E-02

27	cg06322432	10			N_Shore	112289853	0.07 (0.01)	3.37E-06	1.00E+00	5.39E-02
28	cg13153009	8	*ZC3H3*	Body	N_Shelf	144561626	-0.38 (0.08)	3.77E-06	1.00E+00	5.60E-02
29	cg13298389	7				49710144	0.31 (0.07)	3.89E-06	1.00E+00	5.60E-02
30	cg14795409	3				128267611	-0.11 (0.02)	3.99E-06	1.00E+00	5.60E-02
31	cg00193021	4	*SNCA*	5'UTR;1stExon;5'UTR;1stExon;1stExon ;5'UTR;5'UTR	Island	90758120	-0.15 (0.03)	4.24E-06	1.00E+00	5.60E-02
32	cg23827531	3	*FAM107A*	1stExon;5'UTR;5'UTR;1stExon		58563097	0.06 (0.01)	4.35E-06	1.00E+00	5.60E-02
33	cg26917480	17	*ADAP2*	TSS1500	N_Shore	29247893	0.13 (0.03)	4.36E-06	1.00E+00	5.60E-02
34	cg14240060	10				130830949	0.15 (0.03)	4.47E-06	1.00E+00	5.60E-02
35	cg01548142	7			N_Shore	155914873	-0.27 (0.06)	4.60E-06	1.00E+00	5.60E-02
36	cg17203352	17	*WFIKKN2*	TSS200		48912487	-0.08 (0.02)	4.79E-06	1.00E+00	5.60E-02
37	cg03549240	8			S_Shelf	42701169	-0.20 (0.04)	4.89E-06	1.00E+00	5.60E-02
38	cg14950834	19	*SPTBN4;BLVRB*	TSS1500;TSS200	Island	40971830	-0.29 (0.06)	4.96E-06	1.00E+00	5.60E-02
39	cg03012642	2				21346892	-0.10 (0.02)	5.06E-06	1.00E+00	5.60E-02
40	cg17730176	17	*TIAF1;MYO18A*	TSS1500;Body;Body	N_Shelf	27404126	0.17 (0.04)	5.27E-06	1.00E+00	5.68E-02
41	cg22264170	6				9722973	-0.07 (0.02)	5.52E-06	1.00E+00	5.81E-02
42	cg00082729	4				54630659	-0.02 (0.00)	5.83E-06	1.00E+00	5.89E-02
43	cg26393837	11	*PCSK7*	Body	N_Shelf	117099918	0.16 (0.04)	6.01E-06	1.00E+00	5.89E-02
44	cg17989572	17	*RAB5C*	5'UTR;5'UTR	N_Shore	40306700	-0.26 (0.06)	6.02E-06	1.00E+00	5.89E-02
45	cg24751284	14	*APEX1*	Body;Body;Body	S_Shore	20925551	0.12 (0.03)	6.38E-06	1.00E+00	5.89E-02
46	cg02942594	19	*LOC148189*	Body	Island	28284741	-0.21 (0.05)	6.41E-06	1.00E+00	5.89E-02
47	ch.22.46830341F	22				48451677	-0.22 (0.05)	6.67E-06	1.00E+00	5.89E-02
48	cg18051668	17			S_Shelf	55126941	0.17 (0.04)	6.71E-06	1.00E+00	5.89E-02
49	cg07342901	11	*H19;MIR675*	Body;TSS1500	N_Shore	2018724	0.11 (0.02)	6.91E-06	1.00E+00	5.89E-02
50	cg24611315	10				75704459	-0.16 (0.03)	6.94E-06	1.00E+00	5.89E-02

Genes in brackets indicate manual annotation for closest genes using the UCSC genome browser on human GRCh37/hg19 assembly. The record highlighted in red indicates the last methylation site significantly associated with the outcome acccording to Bonferroni correction and the blue highlighted record the last signficant according to false-discovery rate.[5] Cell type heterogeneity is accounted for by adjustment for 6 WBC types according to Houseman[10] (see methods section of main manuscript). EWAS model is same as main reference-based EWAS model (see Supplementary Tables S1-S6) but further adjusted for indicator coded variable "Any maternal smoking during pregnancy (y/n)".

Supplementary Table S33: Change in fat free mass (kg) per percent change in DNA-methylation adj. for maternal smoking during pregnancy (top 50 probes).

Rank	CpG ID	CHR	Gene	UCSC REFGENE GROUP	RELATION TO UCSC CPG ISLAND	Position (bp)	Change (SE) in FFM per % change in DNAm	*P*-value	Bon-ferroni	FDR
1	cg27038634	8	*(LOC101929268)*		N_Shore	49468341	0.13 (0.02)	1.00E-08	4.32E-03	4.32E-03
2	cg08074767	6	*(MLLT4)*			168113741	-0.15 (0.03)	3.27E-08	1.41E-02	6.95E-03
3	cg24332767	3	*C3orf70*	TSS1500	Island	184871524	0.20 (0.04)	4.84E-08	2.09E-02	6.95E-03
4	cg21126338	13	*FARP1*	Body		99084698	0.38 (0.07)	1.16E-07	5.00E-02	1.25E-02
5	cg10135753	19	*BRSK1*	Body	S_Shore	55817121	0.20 (0.04)	1.87E-07	8.06E-02	1.49E-02
6	cg07719679	7	*STEAP4*	TSS200		87936392	0.18 (0.03)	2.08E-07	8.96E-02	1.49E-02
7	cg21525627	12	*(ZDHHC17)*			77149647	0.28 (0.05)	5.42E-07	2.34E-01	3.19E-02
8	cg06376715	1	*TP73*	Body;Body;Body;Body	Island	3634918	0.12 (0.02)	6.60E-07	2.85E-01	3.19E-02
9	cg25827873	8	*(ERICH1)*			593655	-0.11 (0.02)	7.30E-07	3.15E-01	3.19E-02
10	cg01577646	6	*RPS6KA2*	Body;Body		166911121	-0.21 (0.04)	7.40E-07	3.19E-01	3.19E-02
11	cg02393428	12				106582432	0.14 (0.03)	1.25E-06	5.38E-01	3.93E-02
12	cg16312900	17	*SPATA20*	Body	S_Shelf	48628397	0.12 (0.03)	1.59E-06	6.84E-01	3.93E-02
13	cg09229893	14	*BMP4*	TSS1500;TSS1500	S_Shore	54423811	0.12 (0.02)	1.61E-06	6.94E-01	3.93E-02
14	cg26618041	19	*CILP2*	Body	Island	19651867	0.21 (0.04)	1.76E-06	7.59E-01	3.93E-02
15	cg26662102	1	*AJAP1*	Body;Body		4758079	-0.19 (0.04)	1.78E-06	7.70E-01	3.93E-02
16	cg12466919	5	*SQSTM1*	Body;Body;Body	S_Shelf	179251000	0.21 (0.04)	1.80E-06	7.78E-01	3.93E-02
17	cg03810768	2	*EDAR*	TSS200		109606018	0.12 (0.02)	1.94E-06	8.39E-01	3.93E-02
18	cg26995653	2	*(LINC01115(TMEM18))*			858038	0.13 (0.03)	2.00E-06	8.63E-01	3.93E-02
19	cg26432350	3	*NBEAL2*	Body		47040357	0.24 (0.05)	2.09E-06	9.00E-01	3.93E-02
20	cg26721877	5	*STC2*	TSS1500	Island	172757074	0.22 (0.05)	2.11E-06	9.11E-01	3.93E-02
21	cg26360930	8	*DLC1*	Body;Body;Body		12952504	-0.20 (0.04)	2.13E-06	9.19E-01	3.93E-02
22	cg14699734	19			S_Shore	5806164	0.13 (0.03)	2.15E-06	9.28E-01	3.93E-02
23	cg21393818	17				76626270	-0.37 (0.08)	2.15E-06	9.29E-01	3.93E-02
24	cg05822345	11	*B3GAT1*	5'UTR;5'UTR	S_Shore	134257744	-0.23 (0.05)	2.32E-06	1.00E+00	3.93E-02
25	cg26967755	16	*KATNB1*	Body	S_Shelf	57773694	-0.20 (0.04)	2.33E-06	1.00E+00	3.93E-02

26	cg03174228	9	*TTLL11*	Body	N_Shore	124658583	0.10 (0.02)	2.37E-06	1.00E+00	3.93E-02
27	cg01646784	22	*LGALS2*	TSS1500		37976725	0.18 (0.04)	2.61E-06	1.00E+00	3.96E-02
28	cg17511933	2	*GLI2*	Body		121712960	0.57 (0.12)	2.66E-06	1.00E+00	3.96E-02
29	cg12076692	1	*GNB1*	Body		1718852	0.43 (0.09)	2.80E-06	1.00E+00	3.96E-02
30	cg17935297	19	*CILP2*	Body	Island	19651968	0.42 (0.09)	2.92E-06	1.00E+00	3.96E-02
31	cg02747253	14			Island	105662805	-0.32 (0.07)	3.04E-06	1.00E+00	3.96E-02
32	cg16586518	3	*NBEAL2*	Body		47040214	0.18 (0.04)	3.04E-06	1.00E+00	3.96E-02
33	cg25371332	2				10688317	0.21 (0.04)	3.09E-06	1.00E+00	3.96E-02
34	cg02532700	22	*NCF4*	Body;Body		37257404	0.11 (0.02)	3.20E-06	1.00E+00	3.96E-02
35	cg26413162	1				23289818	0.29 (0.06)	3.21E-06	1.00E+00	3.96E-02
36	cg06410191	5	*LVRN*	1stExon	Island	115298920	0.24 (0.05)	3.35E-06	1.00E+00	4.01E-02
37	cg20380470	10			N_Shore	131987945	0.53 (0.11)	3.73E-06	1.00E+00	4.35E-02
38	cg24667304	1	*WDR8*	Body		3550008	0.31 (0.07)	3.89E-06	1.00E+00	4.41E-02
39	cg06318624	2				174913188	0.12 (0.03)	3.98E-06	1.00E+00	4.41E-02
40	cg02381820	2			Island	121493896	0.50 (0.11)	4.29E-06	1.00E+00	4.54E-02
41	cg25901204	15	*ISL2*	TSS1500	N_Shore	76628858	0.22 (0.05)	4.42E-06	1.00E+00	4.54E-02
42	cg04813904	5	*CD180*	Body		66488838	0.15 (0.03)	4.44E-06	1.00E+00	4.54E-02
43	cg13412615	11	*KCNK4*	TSS1500	N_Shore	64057807	-0.19 (0.04)	4.66E-06	1.00E+00	4.54E-02
44	cg01835725	3	*PCBP4*	TSS1500		52002500	0.15 (0.03)	4.72E-06	1.00E+00	4.54E-02
45	cg06369443	1	*KCNQ4*	Body;Body	N_Shore	41303048	0.36 (0.08)	4.84E-06	1.00E+00	4.54E-02
46	cg24301690	3	*KIAA1257*	5'UTR	Island	128712676	0.12 (0.03)	5.08E-06	1.00E+00	4.54E-02
47	cg01706498	3	*KLHL6*	Body		183260375	0.18 (0.04)	5.28E-06	1.00E+00	4.54E-02
48	cg14391016	6	*CCR6*	TSS1500		167523797	0.25 (0.05)	5.33E-06	1.00E+00	4.54E-02
49	cg04269848	2	*AGAP1*	Body;Body		236846527	0.15 (0.03)	5.34E-06	1.00E+00	4.54E-02
50	cg23299109	7	*SLC26A4;LOC286002*	TSS200;Body	N_Shore	107301010	0.17 (0.04)	5.35E-06	1.00E+00	4.54E-02

Genes in brackets indicate manual annotation for closest genes using the UCSC genome browser on human GRCh37/hg19 assembly. The record highlighted in red indicates the last methylation site significantly associated with the outcome acccording to Bonferroni correction and the blue highlighted record the last signficant according to false-discovery rate.[5] Cell type heterogeneity is accounted for by adjustment for 6 WBC types according to Houseman[10] (see methods section of main manuscript). EWAS model is same as main reference-based EWAS model (see Supplementary Tables S1-S6) but further adjusted for indicator coded variable "Any maternal smoking during pregnancy (y/n)".

Supplementary Table S34: Change in fat free mass index (kg/m²) per percent change in DNA-methylation adj. for matern. smoking during pregnancy (top 50).

Rank	CpG ID	CHR	Gene	UCSC REFGENE GROUP	RELATION TO UCSC CPG ISLAND	Position (bp)	Change (SE) in FFMI per % change in DNAm	*P*-value	Bon-ferroni	FDR
1	cg17935297	19	*CILP2*	Body	Island	19651968	0.24 (0.04)	4.42E-08	1.91E-02	1.91E-02
2	cg06437396	6	*(OSTM1)*		N_Shore	108437013	0.12 (0.02)	1.94E-07	8.36E-02	3.38E-02
3	cg26995653	2	*(LINC01115 (TMEM18))*			858038	0.07 (0.01)	3.14E-07	1.36E-01	3.38E-02
4	cg25048701	11	*FOLR1*	5'UTR;TSS1500;5'UTR;5'UTR		71902284	0.26 (0.05)	4.69E-07	2.02E-01	3.38E-02
5	cg08074767	6	*(MLLT4)*			168113741	-0.07 (0.01)	5.29E-07	2.28E-01	3.38E-02
6	cg13718870	9	*BRD3*	5'UTR	Island	136919144	-0.07 (0.01)	5.43E-07	2.34E-01	3.38E-02
7	cg21525627	12	*(ZDHHC17)*			77149647	0.13 (0.03)	5.49E-07	2.37E-01	3.38E-02
8	cg26618041	19	*CILP2*	Body	Island	19651867	0.10 (0.02)	6.68E-07	2.88E-01	3.48E-02
9	cg15914340	2	*(TSSC1)*			3192619	0.30 (0.06)	7.26E-07	3.13E-01	3.48E-02
10	cg01706498	3	*KLHL6*	Body		183260375	0.10 (0.02)	8.42E-07	3.63E-01	3.63E-02
11	cg21126338	13	*FARP1*	Body		99084698	0.17 (0.03)	1.34E-06	5.77E-01	5.22E-02
12	cg02393428	12				106582432	0.07 (0.01)	1.45E-06	6.26E-01	5.22E-02
13	cg19864468	2	*CHCHD5*	3'UTR		113346566	0.16 (0.03)	1.72E-06	7.40E-01	5.69E-02
14	cg26662102	1	*AJAP1*	Body;Body		4758079	-0.09 (0.02)	1.96E-06	8.44E-01	5.97E-02
15	cg00037763	18	*SETBP1*	Body;Body		42281763	-0.12 (0.03)	2.07E-06	8.95E-01	5.97E-02
16	cg04458776	11	*SLC22A6*	TSS1500;TSS1500;TSS1500;TSS1500		62753944	-0.06 (0.01)	2.22E-06	9.58E-01	5.98E-02
17	cg18704658	17			N_Shelf	80300165	0.20 (0.04)	2.53E-06	1.00E+00	6.42E-02
18	cg13345817	15				101695660	0.16 (0.03)	2.72E-06	1.00E+00	6.53E-02
19	cg14524775	1	*ARHGEF2*	Body;Body;Body	Island	155920832	0.39 (0.08)	2.90E-06	1.00E+00	6.58E-02
20	cg07719679	7	*STEAP4*	TSS200		87936392	0.08 (0.02)	3.11E-06	1.00E+00	6.66E-02
21	cg17644856	2				10687962	0.06 (0.01)	3.45E-06	1.00E+00	6.66E-02
22	cg13749070	15	*IGF1R*	Body		99451935	0.36 (0.08)	3.77E-06	1.00E+00	6.66E-02
23	cg24751284	14	*APEX1*	Body;Body;Body	S_Shore	20925551	0.12 (0.03)	3.99E-06	1.00E+00	6.66E-02
24	cg17989572	17	*RAB5C*	5'UTR;5'UTR	N_Shore	40306700	-0.26 (0.06)	4.06E-06	1.00E+00	6.66E-02
25	cg11817057	6			Island	29924202	0.15 (0.03)	4.34E-06	1.00E+00	6.66E-02

26	cg06369443	1	*KCNQ4*	Body;Body	N_Shore	41303048	0.18 (0.04)	4.46E-06	1.00E+00	6.66E-02
27	cg14391016	6	*CCR6*	TSS1500		167523797	0.12 (0.03)	4.56E-06	1.00E+00	6.66E-02
28	cg17421241	1	*PRDM16*	Body;Body	N_Shore	3201953	-0.07 (0.02)	4.61E-06	1.00E+00	6.66E-02
29	cg18282392	4	*GALNT7*	Body		174166207	-0.11 (0.02)	4.65E-06	1.00E+00	6.66E-02
30	cg22213057	7	*PTPRN2*	Body;Body;Body	S_Shore	157697240	-0.16 (0.03)	5.29E-06	1.00E+00	6.66E-02
31	cg02518775	11	*CNIH2*	Body	S_Shore	66046770	0.08 (0.02)	5.44E-06	1.00E+00	6.66E-02
32	cg11475880	X	*IQSEC2*	Body;Body	Island	53283947	0.10 (0.02)	5.64E-06	1.00E+00	6.66E-02
33	cg26401512	1	*ZNF643*	Body		40922773	-0.10 (0.02)	6.09E-06	1.00E+00	6.66E-02
34	cg17259183	10	*NKX6-2*	TSS1500	Island	134599984	0.08 (0.02)	6.32E-06	1.00E+00	6.66E-02
35	cg26360930	8	*DLC1*	Body;Body;Body		12952504	-0.09 (0.02)	6.39E-06	1.00E+00	6.66E-02
36	cg17481047	10			Island	134692852	0.06 (0.01)	6.57E-06	1.00E+00	6.66E-02
37	cg04813904	5	*CD180*	Body		66488838	0.07 (0.02)	6.57E-06	1.00E+00	6.66E-02
38	cg25371332	2				10688317	0.10 (0.02)	6.82E-06	1.00E+00	6.66E-02
39	cg17287326	10	*AVPI1*	5'UTR;1stExon	Island	99446966	0.17 (0.04)	6.90E-06	1.00E+00	6.66E-02
40	ch.8.1234861F	8	*XKR4*	Body		56321773	-0.44 (0.10)	7.05E-06	1.00E+00	6.66E-02
41	cg23577562	4	*RGS12*	Body;Body		3324940	0.27 (0.06)	7.07E-06	1.00E+00	6.66E-02
42	cg24332767	3	*C3orf70*	TSS1500	Island	184871524	0.08 (0.02)	7.13E-06	1.00E+00	6.66E-02
43	cg00271204	5			N_Shore	139142898	0.09 (0.02)	7.16E-06	1.00E+00	6.66E-02
44	cg17459290	1	*LGALS8*	Body;Body;Body;Body		236707000	0.15 (0.03)	7.33E-06	1.00E+00	6.66E-02
45	cg12321193	13	*EDNRB*	TSS1500		78550384	-0.09 (0.02)	7.43E-06	1.00E+00	6.66E-02
46	cg06410191	5	*LVRN*	1stExon	Island	115298920	0.11 (0.02)	7.58E-06	1.00E+00	6.66E-02
47	cg10861407	9	*FSD1L*	TSS1500;TSS1500;TSS1500	N_Shore	108209278	-0.13 (0.03)	7.72E-06	1.00E+00	6.66E-02
48	cg22078907	17	*USP22*	Body		20920756	0.18 (0.04)	7.74E-06	1.00E+00	6.66E-02
49	cg00701992	19	*PNPLA6*	Body;Body;Body;Body;Body	Island	7621749	-0.27 (0.06)	7.85E-06	1.00E+00	6.66E-02
50	cg25554998	11			N_Shore	1227771	-0.07 (0.02)	7.91E-06	1.00E+00	6.66E-02

Genes in brackets indicate manual annotation for closest genes using the UCSC genome browser on human GRCh37/hg19 assembly. The record highlighted in red indicates the last methylation site significantly associated with the outcome acccording to Bonferroni correction and the blue highlighted record the last signficant according to false-discovery rate.[5] Cell type heterogeneity is accounted for by adjustment for 6 WBC types according to Houseman[10] (see methods section of main manuscript). EWAS model is same as main reference-based EWAS model (see Supplementary Tables S1-S6) but further adjusted for indicator coded variable "Any maternal smoking during pregnancy (y/n)".

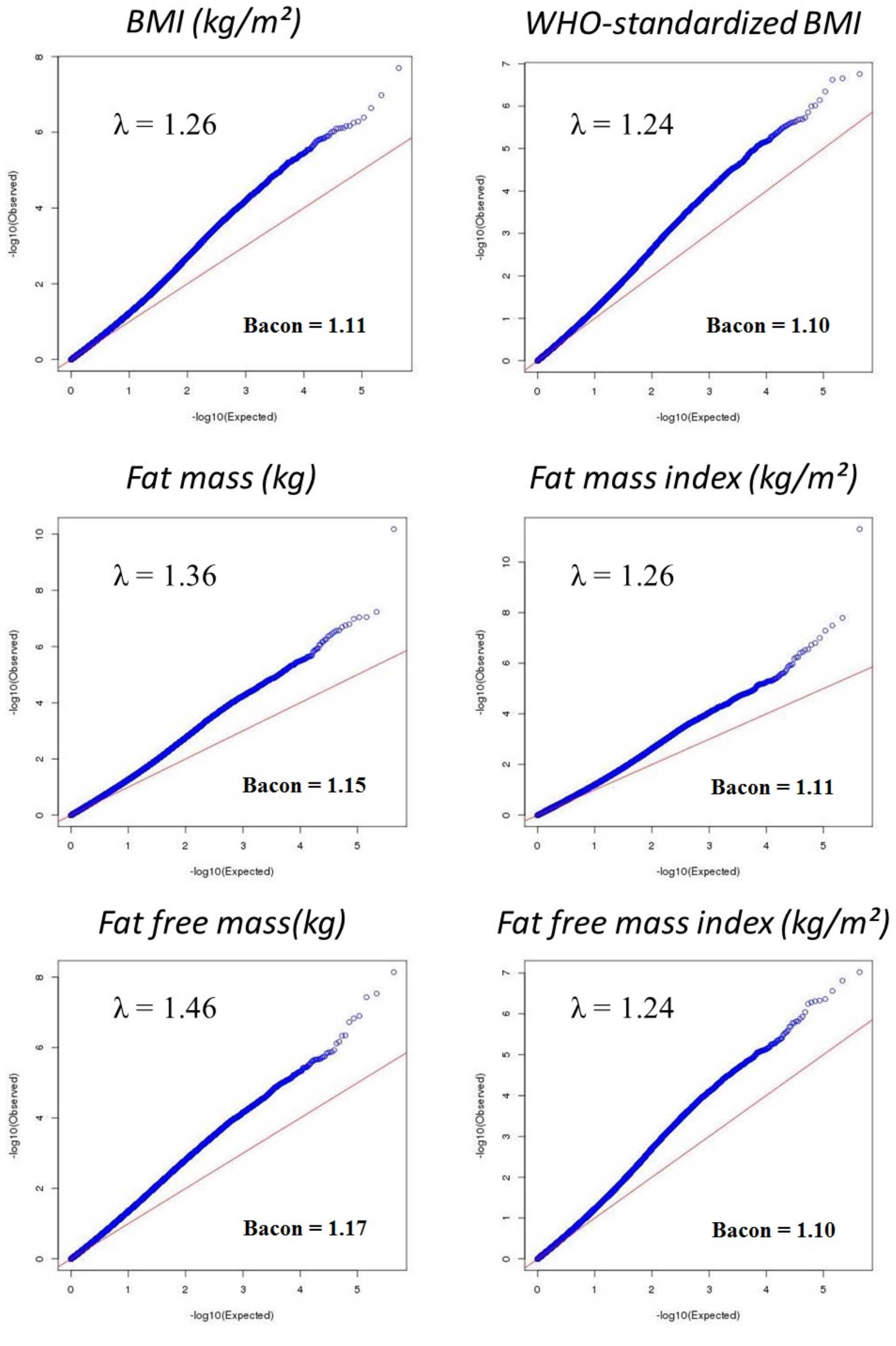

Supplementary Figure S1: Quantile-Quantile Plot of observed vs. expected –log10 *P*-values for each of the 6 outcomes. Q-Q-plots esulting from the regression of respective phenotype on each of 431313 CpG sites and adjustment variables in 374 preschool children. Main blood-referencebased EWAS models in the CHOP study.[10] Lambda (λ) is inflation factor according to genomic control method [8] and Bacon is inflation factor accounting for number of signifcant associations.[9]

References of the online supplement

1. Reimand, J. *et al.* g:Profiler-a web server for functional interpretation of gene lists (2016 update). *Nucleic Acids Res* **44**, W83-9 (2016).
2. Huang, R.C. *et al.* Genome-wide methylation analysis identifies differentially methylated CpG loci associated with severe obesity in childhood. *Epigenetics* **10**, 995-1005 (2015).
3. Wahl, S. *et al.* Epigenome-wide association study of body mass index, and the adverse outcomes of adiposity. *Nature* **541**, 81-86 (2017).
4. Gaunt, T.R. *et al.* Systematic identification of genetic influences on methylation across the human life course. *Genome Biol* **17**, 61 (2016).
5. Benjamini, Y. & Hochberg, Y. Controlling the False Discovery Rate - a Practical and Powerful Approach to Multiple Testing. *Journal of the Royal Statistical Society Series B-Methodological* **57**, 289-300 (1995).
6. Houseman, E.A. *et al.* Reference-free deconvolution of DNA methylation data and mediation by cell composition effects. *BMC Bioinformatics* **17**, 259 (2016).
7. Houseman, E.A., Molitor, J. & Marsit, C.J. Reference-free cell mixture adjustments in analysis of DNA methylation data. *Bioinformatics* **30**, 1431-9 (2014).
8. Devlin, B. & Roeder, K. Genomic control for association studies. *Biometrics* **55**, 997-1004 (1999).
9. van Iterson, M., van Zwet, E.W., Consortium, B. & Heijmans, B.T. Controlling bias and inflation in epigenome- and transcriptome-wide association studies using the empirical null distribution. *Genome Biol* **18**, 19 (2017).
10. Houseman, E.A. *et al.* DNA methylation arrays as surrogate measures of cell mixture distribution. *BMC Bioinformatics* **13**, 86 (2012).
